# Rapid Structural Analysis of Natural Products Using MicroED

**DOI:** 10.1002/smll.202511875

**Published:** 2026-01-23

**Authors:** Jieye Lin, Orel Paz, Johan Unge, Tamir Gonen

**Affiliations:** ^1^ Department of Biological Chemistry University of California, Los Angeles Los Angeles California USA; ^2^ Molecular Biology Institute University of California, Los Angeles Los Angeles California USA; ^3^ Howard Hughes Medical Institute University of California, Los Angeles Los Angeles California USA; ^4^ Department of Chemistry Umeå University Umeå Sweden; ^5^ Department of Physiology University of California, Los Angeles Los Angeles California USA

**Keywords:** crystallography, microcrystal electron diffraction (MicroED), natural products, structural analysis

## Abstract

Structural analysis of natural products remains challenging due to their inherent complexity and limited availability. By solving nine chemically diverse natural products of high priority that have resisted reported 3D structures for years, we demonstrate that microcrystal electron diffraction (MicroED) can systematically and feasibly be applied to natural‐product structure elucidation on samples that were previously unattainable by other structural methods. In addition, using microgram quantities of material and side‐stepping the process to create large single crystals, the success rate is still comparable to other techniques on a general sample using larger amounts of the sample. We suggest that MicroED is not only a necessary technique but also a general and efficient tool for many applications.

## Graphical abstract

The cryoEM method, MicroED used for rapid analysis and structure determination of natural products that remained unattainable by other structural biology methods

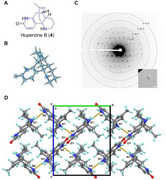

## 1 Introduction

The path toward definitive structural elucidation of natural products may be lengthy and torturous. Literature‐reported structures frequently contain residual errors due to limited data from common techniques or empirical assumptions [1, 2, 3, 4]. The advent of analytical techniques like high‐performance liquid chromatography (HPLC), mass spectrometry (MS), nuclear magnetic resonance (NMR), single‐crystal X‐ray diffraction (SCXRD), etc., significantly enhanced the ability in natural products discovery and characterization, therefore leading to numerous structural revisions reported over the past decade [1, 2, 3, 4].

However, the determination of accurate chemical structures from combined HPLC, MS, and NMR spectroscopic data still requires extensive efforts, ranging from hours to days. Further validation is often relied on chemical resynthesis or SCXRD. Particularly, the SCXRD provides precise atomic details of natural products, including backbone, substitutions, connections, chirality, conformations, etc [3]. For most samples, it is generally pointed out that a minimum dimension of 0.1 mm is needed for a successful SCXRD experiment [5], normally necessitating milligram‐scale samples to identify proper crystallization conditions. It is often unachievable for natural products because of their inherent scarcity and limited purity. Some types of natural products exclusively crystallize in micro‐ or nano‐sized crystals, significantly hindering SCXRD applications in structural elucidation. From experience, the success rate of SCXRD is also limited when amounts larger than ∼50 mg are at hand due to poor crystallization resulting from polymorphism, formation of microcrystals, crystal disorder, or too low a tendency to organize regularly on the scale necessary for SCXRD.

Microcrystal electron diffraction (MicroED) overcame the crystal size limitations [6, 7] by employing electron diffraction to analyze micro‐ or nano‐sized crystals, increasing the overall success rate while enabling samples of limited crystal growth. It requires crystals roughly a billionth of the size needed for conventional SCXRD [8], enabling the direct examination of compounds appearing as amorphous powders or mixtures [8, 9]. Technically, only nanograms of compounds attached to the grid are sufficient for MicroED analysis [8]. MicroED demonstrates an impressive efficiency in data collection and structural elucidation, with a typical dataset collected within 1–2 min and structure solved rapidly in half an hour [8, 10, 11]. These advantages position MicroED as a highly practical tool in natural product discovery, for example, progesterone [8], fischerin [12], lomaiviticin A [13], sinamicin B‐C [14], argyrin‐D [15], hakuhybotrol [16], romidepsin [17], zopalides A‐E [18]. Notably, multiple structures have been directly solved from powder mixtures [8, 9, 19] or HPLC fractions [12, 18, 20] without time‐consuming crystal screening.

Out of 20 selected compounds, whose structures have remained unknown for decades following their discovery, nine natural products resulted in successful structure determination with a good quality resolution of the refined structures. This study provides a detailed structural analysis of those selected natural products. These structures demonstrate: (1) structure determinations can be generally achieved for substantially smaller amounts of samples than required by SCXRD, (2) structure determination using MicroED have a high success rate not only for “easily accessible” targets but also for samples where structure determination have not been published for years, (3) the success rate of about 50% is similar to experiences from SCXRD despite known challenges in the outset, (4) the quality achieved of the refined structures is at least comparable to refined structures from other techniques and (5) bypassing the process of finding the conditions for a large single crystals growth can shorten the structure determination times for future pharmaceutical applications. Thus, while the update of MicroED is growing steadily but cautiously, the advantages of using a general approach with MicroED are suggested.

## 2 Results and Discussion

### 2.1 MicroED Data Collection, Processing, and Refinement

**FIGURE 1:**
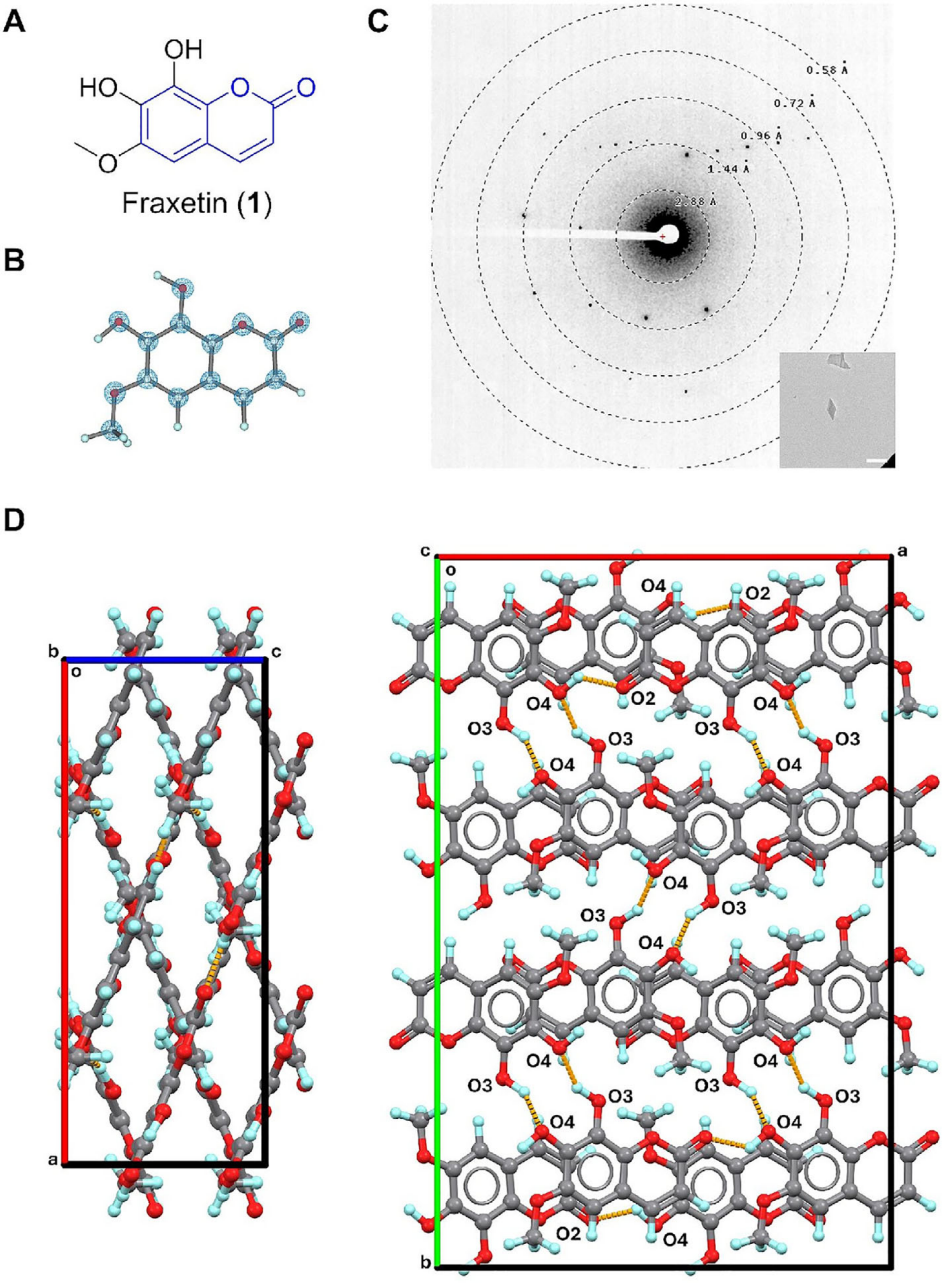
(A) Chemical structure of **1**; (B) MicroED structure of **1**, 2F_o_‐F_c_ density map was shown in a blue mesh at 3σ contour level; (C) Representative diffraction pattern (658 mm) and crystal appearance (SA 5300x) of **1** under TEM. Scale bar: 2 µm; (D) Packing diagram in **1**, viewed along ***b***‐ or ***c***‐ axis. Hydrogen bonding interactions were presented in orange dashed lines.

**FIGURE 2:**
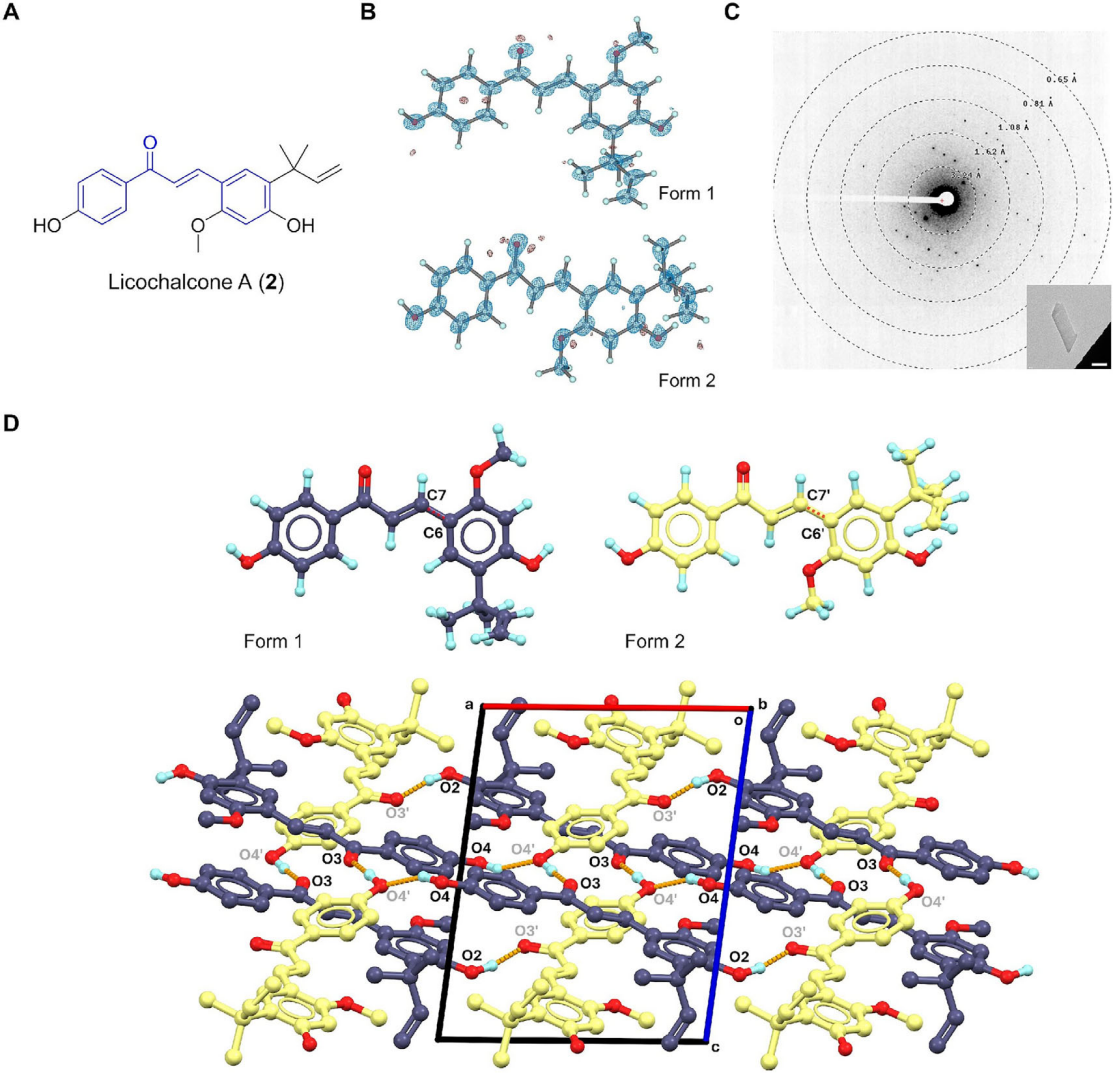
(A) Chemical structure of **2**; (B) MicroED structure of **2**, 2F_o_‐F_c_ density map was shown in a blue mesh at 3σ contour level; (C) Representative diffraction pattern (658 mm) and crystal appearance (SA 5300x) of **2** under TEM. Scale bar: 2 µm; (D) Polymorphic structures and packing diagram in **2**, viewed along ***b***‐axis. Hydrogen bonding interactions were presented in orange dashed lines. Hydrogen atoms not involved in contact were omitted for clarification.

**FIGURE 3:**
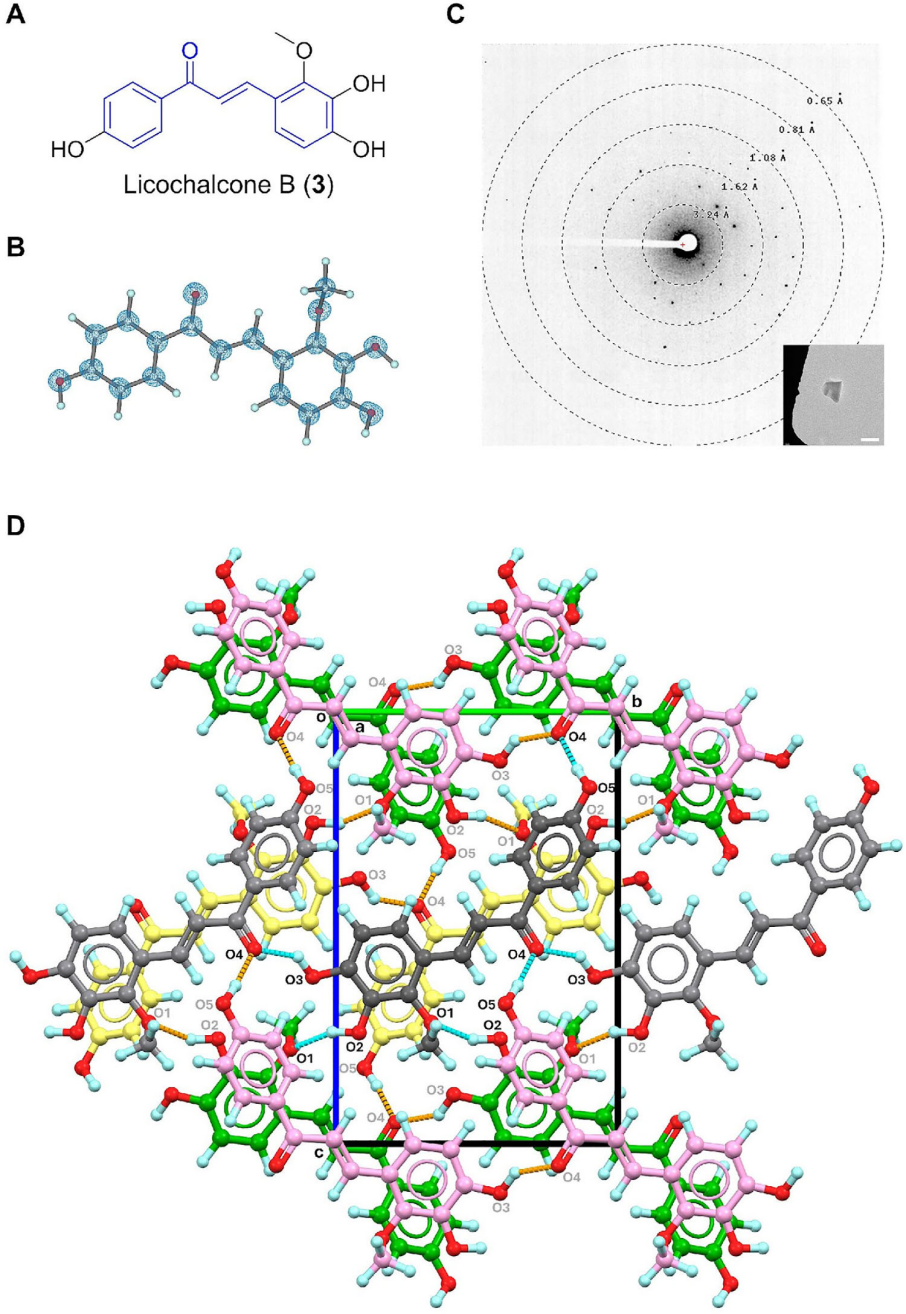
(A) Chemical structure of **3**; (B) MicroED structure of **3**, 2F_o_‐F_c_ density map was shown in a blue mesh at 3σ contour level; (C) Representative diffraction pattern (658 mm) and crystal appearance (SA 5300x) of **3** under TEM. Scale bar: 2 µm; (D) Packing diagram in **3**, viewed along the ***a***‐axis. Hydrogen bonding interactions were presented in orange and cyan dashed lines. Molecules were colored by their symmetries.

**FIGURE 4:**
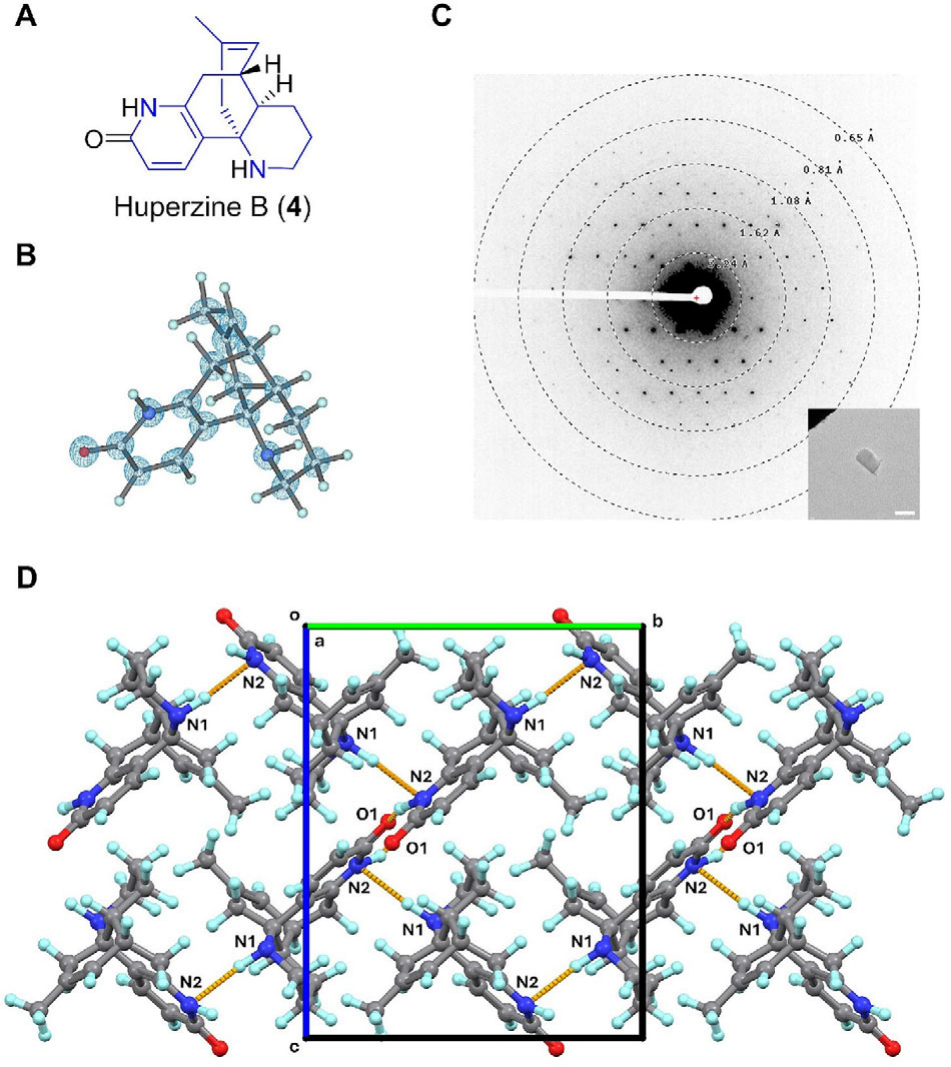
(A) Chemical structure of **4**; (B) MicroED structure of **4**, 2F_o_‐F_c_ density map was shown in a blue mesh at 3σ contour level; (C) Representative diffraction pattern (658 mm) and crystal appearance (SA 5300x) of **4** under TEM. Scale bar: 2 µm; (D) Packing diagram in **4**, viewed along the ***a***‐axis. Hydrogen bonding interactions were presented in orange dashed lines.

**FIGURE 5:**
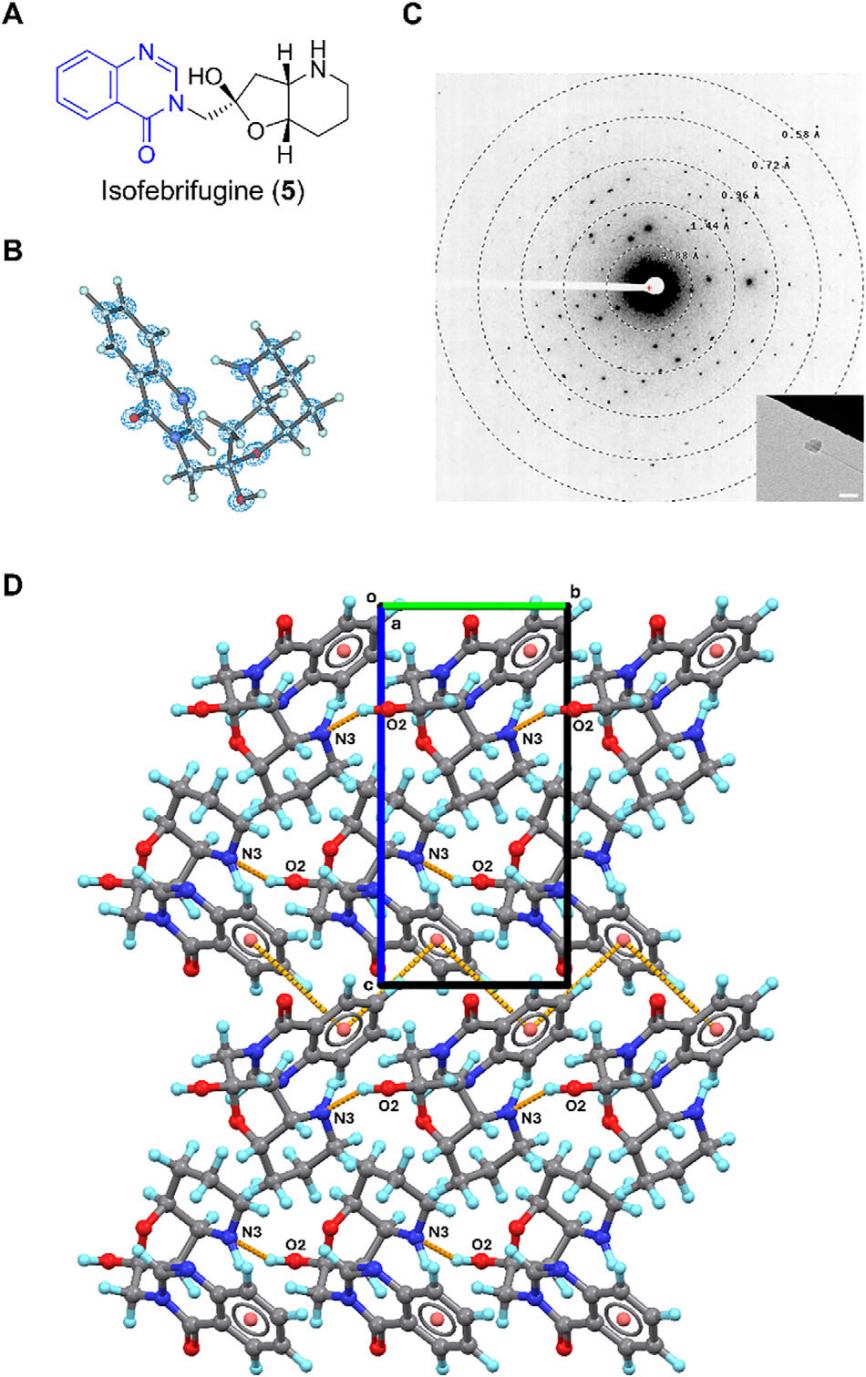
(A) Chemical structure of **5**; (B) MicroED structure of **5**, 2F_o_‐F_c_ density map was shown in a blue mesh at 3σ contour level; (C) Representative diffraction pattern (658 mm) and crystal appearance (SA 5300x) of **5** under TEM. Scale bar: 2 µm; (D) Packing diagram in **5**, viewed along the ***a***‐axis. π‐π and hydrogen bonding interactions were presented in orange dashed lines.

**FIGURE 6:**
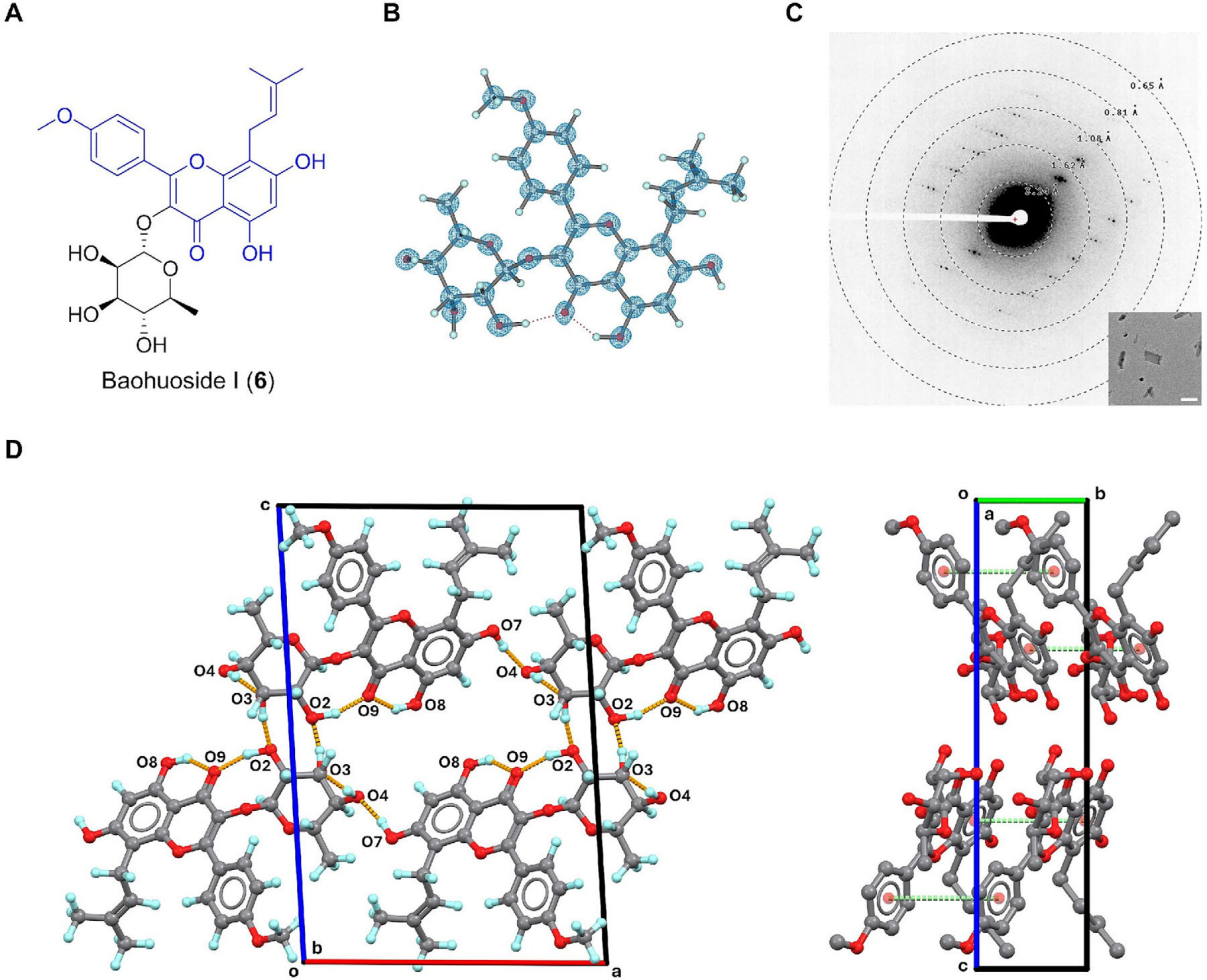
(A) Chemical structure of **6**; (B) MicroED structure of **6**, 2F_o_‐F_c_ density map was shown in a blue mesh at 3σ contour level; (C) Representative diffraction pattern (658 mm) and crystal appearance (SA 5300x) of **6** under TEM. Scale bar: 2 µm; (D) Packing diagram in **6**, viewed along ***a***‐ or ***b***‐axis. Hydrogen bonding interactions were presented in orange dashed lines. π‐π interactions were presented in green dashed lines. Hydrogen atoms not involved in contact were omitted for clarification.

**FIGURE 7:**
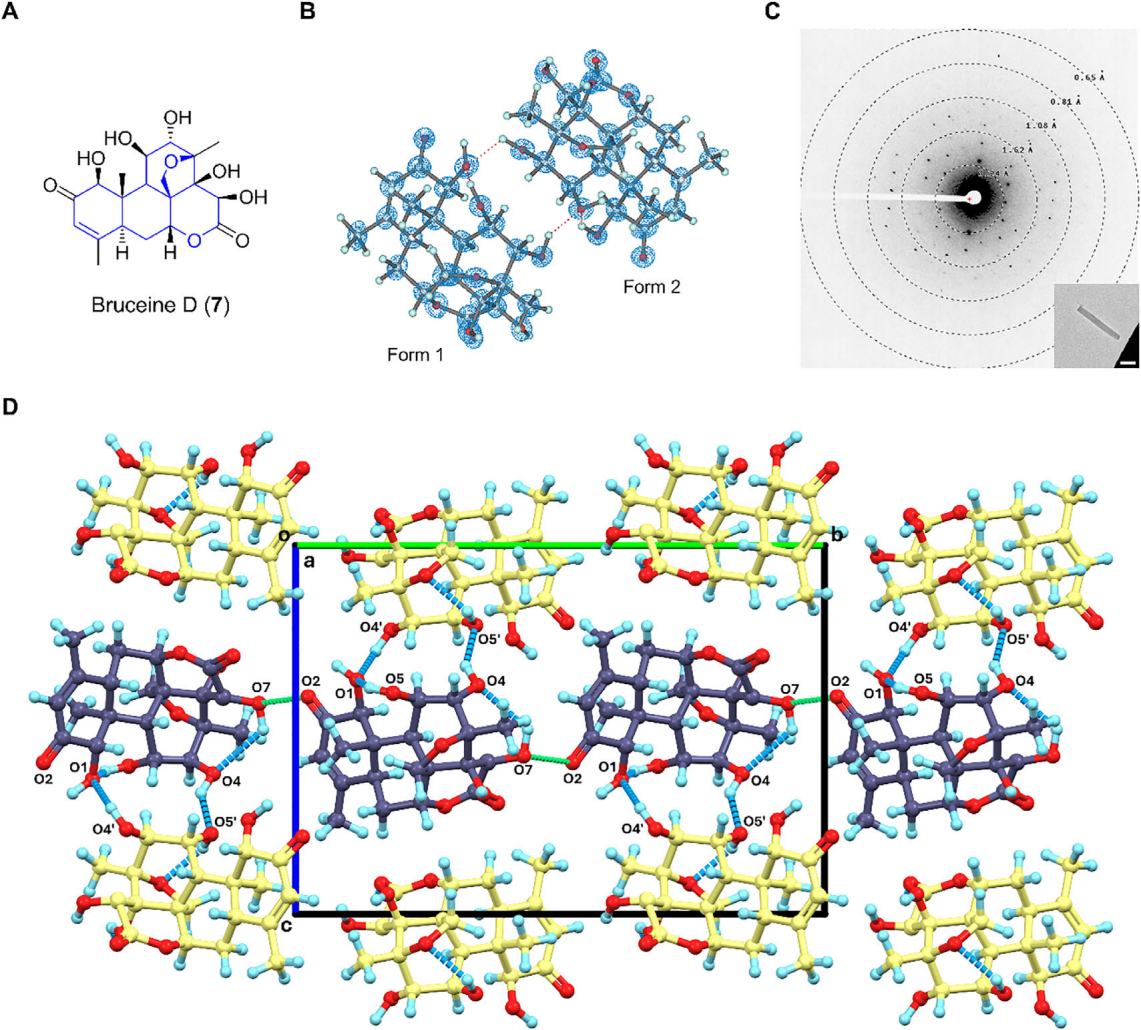
(A) Chemical structure of **7**; (B) MicroED structure of **7**, 2F_o_‐F_c_ density map was shown in a blue mesh at 3σ contour level; (C) Representative diffraction pattern (658 mm) and crystal appearance (SA 5300x) of **7** under TEM. Scale bar: 2 µm; (D) Packing diagram in **7**, viewed along the ***a***‐axis. Forms 1 and 2 were presented in purple and yellow, respectively. Hydrogen bonding interactions were presented in blue dashed lines.

**FIGURE 8:**
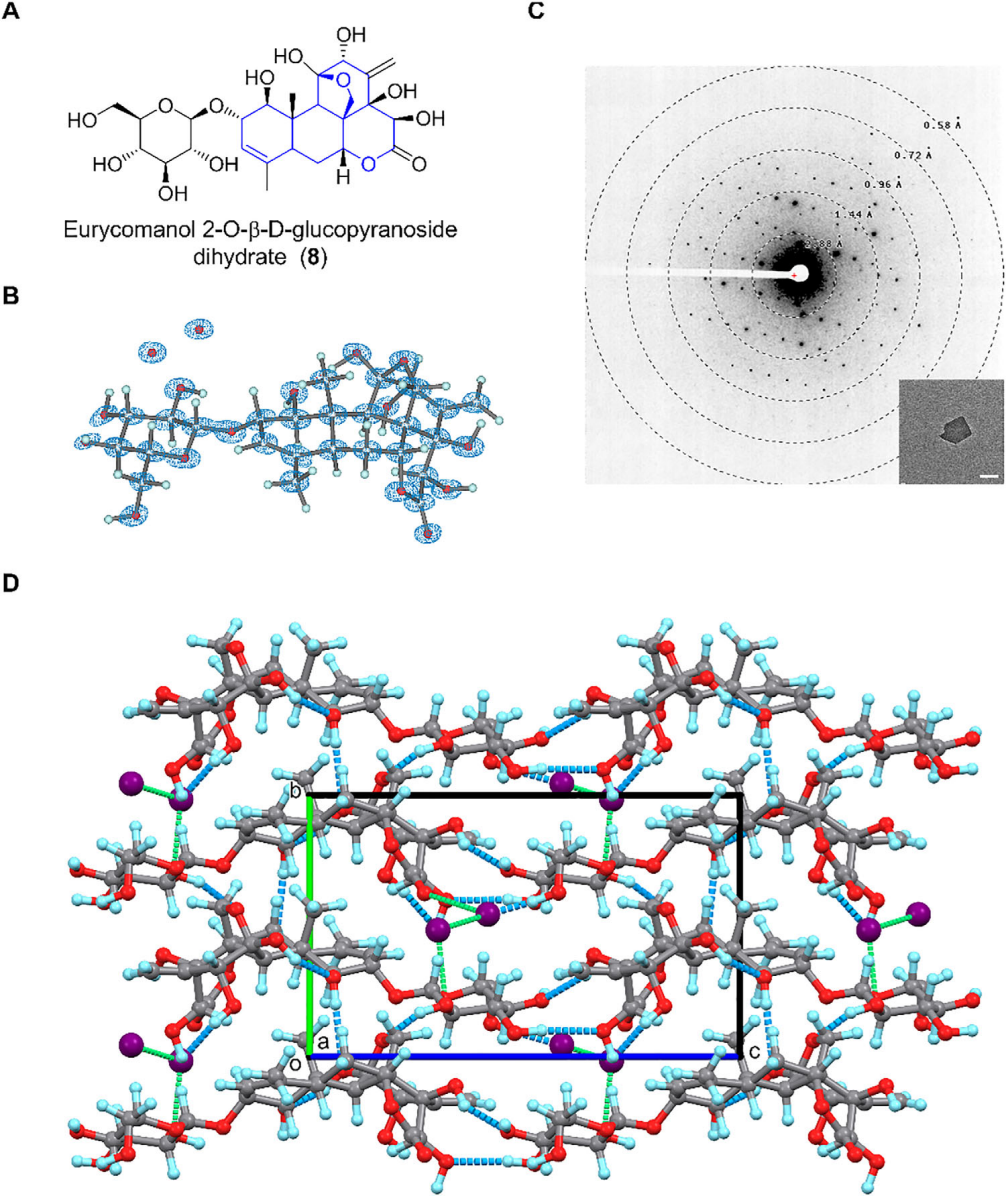
(A) Chemical structure of **8**; (B) MicroED structure of **8**, 2F_o_‐F_c_ density map was shown in a blue mesh at 3σ contour level; (C) Representative diffraction pattern (658 mm) and crystal appearance (SA 5300x) of **8** under TEM. Scale bar: 2 µm; (D) Packing diagram in **8**, viewed along the ***a***‐axis. Hydrogen bonding interactions were presented in blue and green dashed lines. Water molecules were colored in purple balls.

**FIGURE 9:**
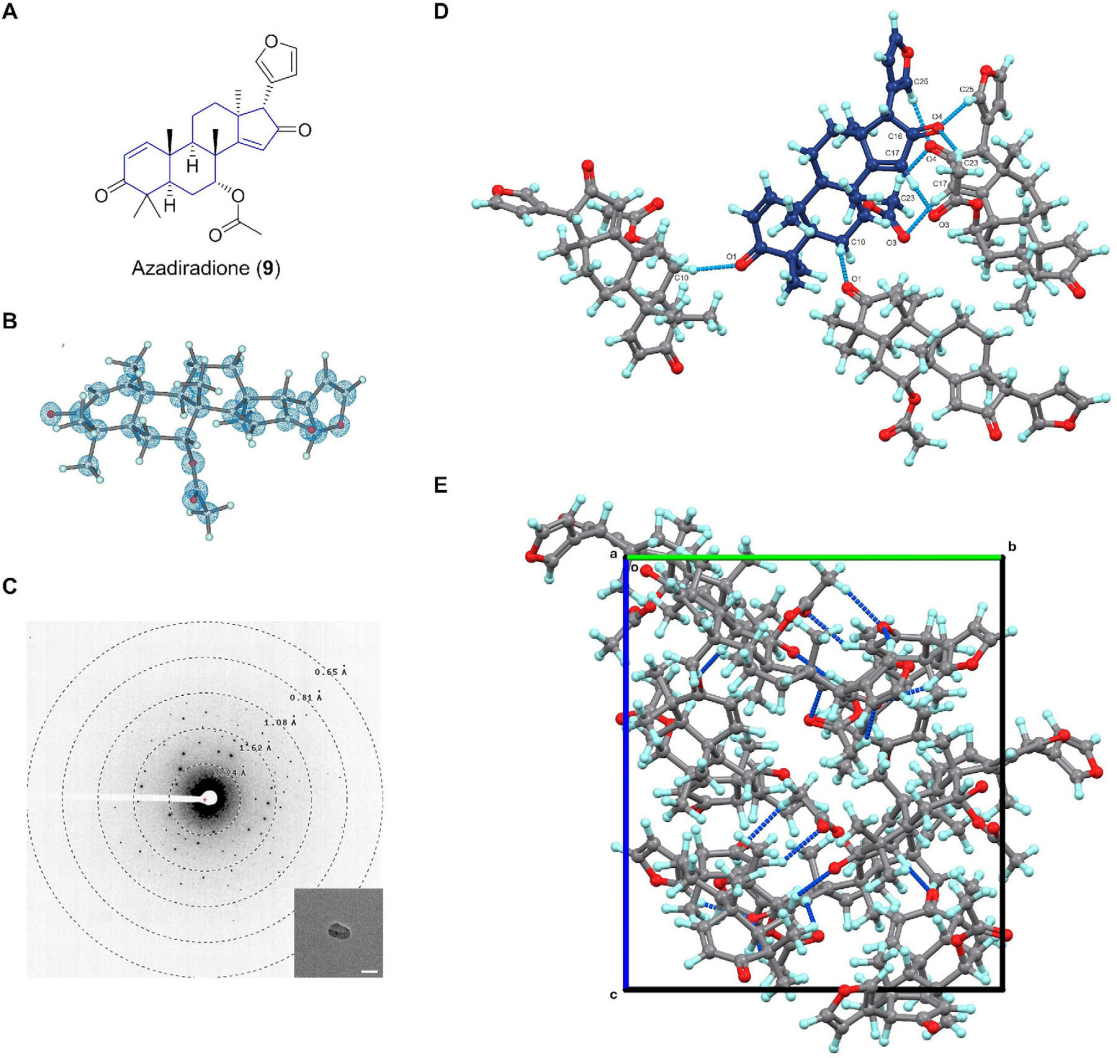
(A) Chemical structure of **9**; (B) MicroED structure of **9**, 2F_o_‐F_c_ density map was shown in a blue mesh at 3σ contour level; (C) Representative diffraction pattern (658 mm) and crystal appearance (SA 5300x) of **9** under TEM. Scale bar: 2 µm; (D) Weak C─H···O hydrogen bonding interaction for one molecule of **9**. (E) Packing diagram in **9**, viewed along the ***a***‐axis. Hydrogen bonding interactions were presented in blue dashed lines.

Less than 1 mg powder samples for **1**–**9** each were used in MicroED analysis. To enhance the success rate of structural elucidation, approximately 20 datasets were collected for each sample. The selected thin crystals showing light contrast to the carbon film have resolutions higher than 0.9 Å in initial diffraction tests, and crystal sizes ranging from ∼0.3 to 2 µm (Figures 1, 2, 3, 4, 5, 6, 7, 8, 9). Their eucentric heights were manually calibrated to maintain them within view of the selected area aperture during continuous rotation. MicroED movies were recorded using a CMOS Ceta‐D camera at a constant rotation speed of 1°/s and exposure time of 1 s per frame (See Section 3), consuming 2 mins for each dataset. MicroED data were saved in mrc format and rapidly processed using mrc2smv [21], XDS [22], XSCALE [23], XDSCONV [23] (< 3 min/dataset). High resolution limits ranging from 0.60 to 0.86 Å were used to yield completeness from 85% to 100% (See Section 3; Tables S1–S9). Crystal structures were *ab initio* solved by SHELXT/D [24, 25] and refined by SHELXL [26], requiring ∼30 mins per dataset. Final R1 values ranged from 12.23 to 17.85%, indicating satisfactory data quality (See Tables S1–S9). Heavier atoms were resolved from density maps and demonstrated consistency with the proposed chemical structures. Hydrogen atoms were refined using a combination of free and restrained approaches (See Section 3).

### 2.2 Fraxetin (1)

Fraxetin (**1**) was first characterized as an aglucon hydrolyzed from fraxin, initially isolated from the barks of *Fraxinus excelsior* in 1928 [27]. It exhibits multiple biological activities, including antioxidative, anticancer, and antidysenteric, etc [28, 29, 30]. The chemical structure of **1** consists of a coumarin backbone [31], substituted by two ─OH and one ─OCH_3_ groups (Figure 1A). Detailed 3D structural information remained unknown until the 0.63 Å MicroED structure was solved (Figure 1B). The crystal structure of **1** revealed a planar shape of the coumarin core and the attached exocyclic ─OH and ─OCH_3_ groups. The ─OCH_3_ group positions away from ─OH groups because of structural hindrance. Similar conformations were observed in related compounds such as fraxidin (CCDC entry: TUSQEG) [32], 7‐hydroxy‐6,8‐dimethoxycoumarin (MIKZIP) [33], etc. (Scheme S1). **1** pack in the space group *F*dd2, exhibiting parallel and intercrossed molecular sheets (Figure 1D). Crystal packing is stabilized by recurring O4─H···O2 hydrogen bonds (2.633 Å) on the diagonal directions of the ***ac***‐plane, with additional O3─H···O4 hydrogen bonds (2.780 Å) and π···π interactions reinforcing connections between two adjacent layers.

### 2.3 Licochalcone A (2)

Licochalcone A (**2**) is a chalconoid first isolated from the root of *Glycyrrhiza glabra* in 1975 [34]. It possesses biological activities like anticancer, anti‐inflammatory, antibacterial, antimalarial, etc [35, 36, 37]. The chemical structure of **2** comprises a chalconoid backbone [38] with substitutions of ─OH, ─OCH_3_, and 1,1‐dimethyl‐2‐propenyl groups on two phenyl rings (Figure 2A). MicroED resolved its structure at 0.75 Å resolution in the *P*‐1 space group, revealing two conformers (forms 1 and 2) within the asymmetric unit (Figure 2B). These conformers primarily differ in the C6─C7/C6′─C7′ bond (Figure 2D): the form 1 shows a C8─C7─C6─C1 torsion angle of 173.1° (*trans*), while the C8′─C7′─C6′─C1′ torsion angle of ‐3.25° (*cis*). In form 1, two phenyl rings lie almost coplanar to the plane defined by C6, C7, C8, C9, and C10 atoms with only 5° distortions; in form 2, two phenyl rings show a more significant distortion of around 25° distortions. The crystal lattice is connected via a complicated hydrogen bonding network between forms 1 and 2 (Figure 2D). Each molecule of form 1 interacts with three molecules of form 2, donating two hydrogen bonds (O2─H···O3′, 2.601 Å; O4─H···O4′, 2.748 Å) and accepting one hydrogen bond (O4′─H···O3, 2.581 Å); The O2′ atom in form 2 doesn't participate in hydrogen bonding therefore resulting the disrupted crystal growth along ***b***‐axis.

### 2.4 Licochalcone B (3)

Licochalcone B (**3**), another chalconoid isolated from *Glycyrrhiza glabra* root in 1975 [34], exhibits biological activities including anti‐inflammatory, antioxidant, anticancer, etc [39]. Similar to **2**, it features a chalcone backbone [38] substituted with three ─OH and one ─OCH_3_ groups on the phenyl rings (Figure 3A). The MicroED structure of **3**, resolved at 0.70 Å resolution, showed a comparable geometry to form 1 of **2**, differing primarily in the torsion angle of one phenyl ring and other exocyclic substitutions (Figure 3B; Figure S1). The dense crystal packing of **3** adopts the symmetry in the *P*2_1_/c space group, forming interconnected layers of molecules. Three hydroxyl groups (O2, O3, O5 atoms) serve as three hydrogen donors, while the rest methoxy (O1) and carbonyl (O4) groups act as acceptors for three hydrogen bonds (Figure 3D), i.e., O2─H···O1 (2.803 Å), O3─H···O4 (2.673 Å), O5─H···O4 (2.719 Å). Weak π···π stacking interactions (4.044 and 4.296 Å) between phenyl groups help stabilize the crystal packing.

### 2.5 Huperzine B (4)

Huperzine B (**4**) is a lycodine‐type alkaloid isolated from *Huperzia serrata* in 1986 [40]. It is a promising treatment for Alzheimer's disease due to its inhibitory activity against cholinesterase (AChE) [41, 42]. The chemical structure of **4** resembles that of lycodine, huperradine A (CCDC entry: JOCTEE) [43] and lycophlegmarinine D (GEJBEG) [44], differing mainly in minor backbone distortions (Figure 4A). Comparing the 0.67 Å MicroED structure of **4** to huperradine A [43] showed a consistent backbone geometry (Figure 4B; Figure S2A), except one ─NH group is *axial* in **4** but *equatorial* in huperradine A. The fused ring system in **4** maintains a rigid conformation from the solid state to the protein‐bound state (PDB entry: 1GPN; Figure S2B) [45]. Only minimal ring distortions were observed in exocyclic ─C═O and ─CH_3_ groups. Crystal structure of **4** packs in *P*2_1_2_1_2_1_ space group and is extended by repetitive N2─H···O1 (2.816 Å) along ***a***‐axis, and weak N1─H···N2 (3.562 Å) along ***b***‐ and ***c***‐axes (Figure 4D). When bound to acetylcholinesterase, the O1 and N2 atoms form hydrogen bonds with Gly117 and Gly118 residues [45], consistent with interactions observed in the solid state.

### 2.6 Isofebrifugine (5)

Isofebrifugine (**5**) is a quinazolinone‐type alkaloid first isolated from *Dichroa febrífuga* in 1950 [46]. It exhibits antimalarial and antiplasmodial properties [47] with notably lower toxicity compared to its isomer febrifugine [48, 49]. The chemical structure of **5** was initially proposed in 1950 and later revised in 1999 [50, 51]. Both chemical structures of **5** and febrifugine contain a quinazolinone backbone, but **5** has a hemiketal‐like ring cyclized by fusing a 3‐hydroxypiperidine ring with a ─C═O group (Figure 5A) [46, 50, 51]. The crystal structure of febrifugine was solved by SCXRD in 2016 (CCDC entry: WIDQOS) [52], showing a *trans*‐ planar geometry. In contrast, the MicroED structure **5** solved at 0.60 Å adopts a *cis*‐ form and “L‐shaped” conformation (Figure 5B). Reaching this folded conformation from febrifugine requires approximately 180° rotation of the 3‐hydroxypiperidine ring, potentially forming a hydrogen bond between ─OH and ─C═O group, considering the preferred stereochemistry at the stereochemical center. The crystal packing in **5** follows the symmetry of the *P*2_1_ space group, a lower symmetry than febrifugine (*P*bca space group), since fewer chemical substitutions can contribute to the hydrogen bonding network in **5**. The crystal lattice of **5** is constructed via weak interactions (Figure 5D), i.e., the O2─H···N3 (2.751 Å) hydrogen bond and *zig‐zagged* T‐shaped π‐π interactions along the ***b***‐axis.

### 2.7 Baohuoside I (6)

Baohuoside I (**6**), also known as icariside II, is an icaritin‐type flavonoid first isolated from *Epimedium davidii Franch* in 1988 [53]. **6** demonstrates anti‐inflammatory, immunosuppressant, and anticancer activities [54, 55]. Its chemical structure comprises an icaritin skeleton, common among related icaritin‐type flavonoids [56] like icariin, icariside I, epimedin A‐C, etc. (Figure 6A), but uniquely substituted with an L‐rhamnoside moiety. Variations among these flavonoids primarily lie in the glycan moieties, for example, **6** (L‐rhamnoside), icariin (L‐rhamnoside and D‐glucoside), and icariside I (D‐glucoside) [56]. The crystal structures of the above compounds remain largely unexplored; only a SCXRD structure of icariin (CCDC entry: KUNPAM) [57] was deposited in literature. Comparing the MicroED structure of **6** solved at 0.73 Å to icariin (Figure 6B; Figure S3) reveals a conserved backbone, but significant differences in the conformation of exocyclic ─OCH_3_ and prenyl groups, and ring distortions in L‐rhamnoside (Figure S3). The Cremer‐Pople analysis [58] indicates a remarkably distorted ^2^ *S*_O_ L‐rhamnose ring in **6**, differing from the typical ^1^ *C*_4_ ring conformation found in icariin (Figure 6B; Figure S3). The former ring distortion tentatively arose from an intramolecular hydrogen bond O2─H···O9 (2.706 Å). Crystal packing of **6** involves multiple hydrogen bonds (Figure 6D), including two intramolecular hydrogen bonds, i.e., O2─H···O9 (2.706 Å, along ***a***‐axis), O8─H···O9 (2.636 Å, along ***a***‐axis); three intermolecular hydrogen bonds, i.e., O3─H···O2 (2.604 Å, along ***c***‐axis), O4─H···O3 (2.685 Å, along ***a, b***‐axes), O7─H···O4 (2.629 Å, along ***a, b***‐axes). Additionally, weak π···π stacking interactions between two phenyl groups of the icaritin backbone (4.680 Å along the ***b***‐axis) help stabilize the crystal packing.

### 2.8 Bruceine D (7)

Bruceine D (**7**) is a quassinoid first isolated from *Brucea amarissima* in 1967 [59]. **7** has various biological functions, including antimalarial, antiplasmodial, anticancer, etc [47, 60]. Like the other related compounds, such as bruceine A‐M and yadanziolide S [60], the chemical structure of **7** features a tetracyclic skeleton with a fused lactone ring (Figure 7A), differing primarily in exocyclic substitutions and chirality. Previous NMR studies determined the 2D chemical structure of **7**; however, its 3D atomic structure remained unclear. The MicroED structure of **7** solved at 0.78 Å in the *P*2_1_ space group, reveals two independent molecules in the asymmetric unit (Figure 7B). Overlaying forms 1 and 2 demonstrates a comparable backbone geometry, yet notable differences in the conformations of exocyclic ─OH groups and rings (Figure S4). **7** shows a more rigid conformation compared to the structure of yadanziolide S (CCDC entry: YABPIB) [61], and the latter displays greater flexibility in exocyclic ─OH and ─CH_3_ groups. The crystal lattice of **7** is characterized by repetitive molecular layers (Figure 7D). Due to the data quality of **7**, not all the H atoms can be unbiasedly located from the difference density map. From the current model, forms 1 and 2 pack in pairs via intermolecular hydrogen bonds, like O4′─H···O1 (3.339 Å, along ***b***, ***c***‐axes) and O4─H···O5′ (2.888 Å, along ***c***‐axis). Form 1 is potentially extended via a bifurcated O7─H···O2 hydrogen bond (2.827 Å, along ***b***‐axis), while it is missing in form 2. Five intramolecular hydrogen bonds, i.e., O5─H···O1 (2.821 Å), O7─H···O4 (3.205 Å), O8─H···O6 (2.901 Å), O5′─H···O9′ (2.839 Å), O8′─H···O6 (2.850 Å), extend the crystal packing of **7** along ***a***‐ and ***b***‐axes. It is found that the interactions along the ***c***‐axis are severely weaker than along the ***a***‐ and ***b***‐axes, potentially contributing to uneven crystal growth and smaller crystal sizes.

### 2.9 Eurycomanol 2‐O‐β‐D‐glucopyranoside Dihydrate (8)

Eurycomanol 2‐*O*‐β‐D‐glucopyranoside dihydrate (**8**) is a quassinoid identified from the butanol extraction of *Eurycoma longifolia* roots in 1989 [62]. It exhibits antimalarial and antiplasmodial biological activities [47]. The chemical structure of **8** shares a similar tetracyclic backbone with **7** but differs in the fused lactone ring (Figure 8A). Structural comparison of the 0.73 Å MicroED structure of **8** (Figure 8B) and related SCXRD structures like eurycomanone (CCDC entry: VOQPIB) [63], ailanthone (BITWIK10) [64], pasakbumin C (EZISUC) [65], reveals similar backbone geometry (Figure S5). The D‐glucoside moiety adopts a standard ^4^ *C*_1_ chair conformation and is covalently connected via a β‐glycosidic bond with *φ* (C19─C18─O8─C1: 145.41°; O9─C18─O8─C1: −93.45°) and *ψ* (C18─O8─C1─C2: −164.71°; C18─O8─C1─C6: 70.65°). Crystal packing of **8** follows the symmetry of the *P*2_1_ space group, therefore layers of molecules pack reversely along the ***b***‐axis and repetitively extend along the ***a***‐ and **c**‐axes (Figure 8D). Current data quality does not allow for the direct localization of all H atoms from the difference density map. The refined model suggests each molecule interacts with up to eight neighboring molecules via at least three different intermolecular and one intramolecular hydrogen bonds (Figure S6), i.e., O4─H···O12 (2.874 Å), O6─H···O7 (2.676 Å), O11─H···O3 (3.312 Å), O10─H···O5 (2.863 Å). Additionally, two water molecules between layers of **8** (although the hydrogen atoms were not seen from maps), likely form at least six water bridges to reinforce the crystal packing, i.e., OW1···OW2 (2.689 Å), OW1···O3 (2.759 Å), OW1···O10 (2.806 Å), OW1···O14 (2.798 Å), OW2···O13 (2.713 Å).

### 2.10 Azadiradione (9)

Azadiradione (**9**) is a limonoid first identified in the seed oil of *Melia azadirachta* in 1967 [66]. **9** exhibits antibacterial, antimalarial, and antifeedant activities [67, 68]. The chemical structure of **9** includes an alkane‐based skeleton commonly found in limonoid‐type compounds [69] such as azadirone (CCDC entry: TERNAG) [70] and 6β‐acetoxyazadirone (LIYYIB) [71], but differs in the exocyclic substitutions (Figure 9A). The 0.86 Å MicroED structure of **9** showed strong backbone similarity to azadirone, with minor ring distortions around C1 and C16 atoms. A notable structural difference is the ∼50° furan ring's rotation in **9** and azadirone (Figure 9B; Figure S7A). The conformation of the exocyclic groups had minimal impact on overall backbone geometry, as similarly observed in 6β‐acetoxyazadirone (Figure S7B) [71]. Consistent with other limonoids, the crystal packing in **9** is complex, exhibiting high symmetry (*P*4_3_2_1_2), which is constructed via repetitive C─H···O hydrogen bonds (Figure 9D). Each molecule acts as both donor and acceptor, repetitively interacting with three other molecules via four hydrogen bonds, i.e., C25─H···O4 (3.352 Å), C23─H···O4 (3.335 Å), C17─H···O3 (3.338 Å) and C10─H···O1 (3.417 Å).

## 3 Methods

### 3.1 Materials

Commercial compounds **1**, **2**, **4**, **5**–**9** were purchased from InvivoChem. Commercial compounds **3** and **6** were purchased from MedChemExpress. All the compounds were used as received without recrystallization.

### 3.2 Sample Preparation

The 400‐mesh, 3.05 mm O.D copper grid (prod.#: G400; Ted Pella, Inc.) was coated with a collodion support film, followed by continuous carbon sputtering using EM ACE600 sputter coater (Leica). Grids were subsequently treated with 15 mA negative glow‐discharge plasma for 45 s using PELCO easiGlow (Ted Pella Inc.). Approximately 1 mg or less of each powdery sample was transferred and mixed with a TEM grid separately, followed by 30 s gentle shake in a glass vial. A TEM grid containing the tested sample was clipped with an autogrid ring and a C‐clip for the subsequent MicroED analyses.

### 3.3 MicroED Data Collection

The prepared autogrids were loaded into a Thermo Fisher Talos Arctica Cryo‐TEM (200 kV, ∼0.0251 Å). A CMOS Ceta‐D camera (4096 × 4096 pixels) and EPU‐D software (Thermo Fisher) facilitated screening and data collection [8]. Thin microcrystals that appeared with light contrast against the carbon film were selected under imaging mode (SA 3400x). Their eucentric heights were manually calibrated to maintain the crystals inside the beam during continuous rotation. MicroED data were collected in the diffraction mode with a sample‐detector distance of 658 mm and a microprobe spot size of 11 under parallel illumination. The parallel beam (∼45.2% intensity) was tuned using a 500 nm diffraction grating replica (prod.# 673; Ted Pella, Inc.) and 70 µm objective aperture at the back focal plane. A 100 µm selected area (SA) aperture defined a beam area of approximately 2.6 µm width. The electron dose rate was ∼0.01 e^−^/Å^2^·s. Data collection typically involved a constant rotation rate of 1°/s over an angular wedge of 120° from −60° to +60°, with 1 s exposure time per frame, resulting in a total electron dose of ∼1.2 e^−^/Å^2^ per dataset.

### 3.4 MicroED Data Processing

MicroED movies were saved in mrc format and converted to smv images using the mrc2smv software (https://cryoem.ucla.edu/microed) [21]. Diffraction spots were picked, indexed, and integrated using XDS [22]. The resolution was generally cut at the I/sigma of 1.0, CC_1/2_ larger than 30%, R_meas_ less than 100%. Multiple datasets were merged to achieve more than 85% completeness using XSCALE [23]. Intensity data were converted to SHELX hkl format using XDSCONV [23]. The heavier atoms were ab initio solved by SHELXT/D [24, 25]. Hydrogen atoms of hydroxyl and amine groups were refined from corresponding Fo‐Fc difference maps; other Hydrogen atoms were refined on their geometrically calculated positions of C─H═1.120 Å (methine), 1.110 Å (methylene),1.080 Å (methyl), or 1.100 Å (aryl) [26].

## 4 Conclusion

Natural product structures may pose challenges for conventional structural analysis techniques, underscoring the necessity for an additional and more general approach to handle difficulties such as impure samples, limited availability of the sample, or imperfect crystallization. In this study, we successfully utilized MicroED to determine the crystal structures of nine natural products whose 3D atomic structures had remained elusive for decades. From powder to final structure, MicroED shows efficiency in data collection and structural solution, with typical data collected within 1–2 min and structure solved rapidly in half an hour. The resolved structures complement the emptiness of the research in literature and facilitate detailed structural analysis of their constitution, skeleton, conformation, packing, etc., laying robust foundations for future pharmaceutical applications.

## Conflicts of Interest

The authors declare no conflicts of interest.

## Supporting information




**Supporting File**: smll72428‐sup‐0001‐SuppMat.docx.

## Data Availability

The data that support the findings of this study are available in the supplementary material of this article.

## References

[1] S.‐M. Shen , G. Appendino , and Y.‐W. Guo , “Pitfalls in the Structural Elucidation Of Small Molecules. A Critical Analysis Of A Decade Of Structural Misassignments Of Marine Natural Products,” Natural Product Reports 39 (2022): 1803–1832, 10.1039/D2NP00023G. 35770685

[2] P. D. Brown and A. L. Lawrence , “The Importance Of Asking “How And Why?” In Natural Product Structure Elucidation,” Natural Product Reports 34 (2017): 1193–1202, 10.1039/C7NP00025A. 28850146

[3] J.‐G. Song , W.‐C. Ye , and Y. Wang , “Advanced Crystallography For Structure Determination Of Natural Products,” Natural Product Reports 42 (2025): 429–442, 10.1039/D4NP00071D. 39898652

[4] S. Berger and D. Sicker , Classics in Spectroscopy: Isolation and Structure Elucidation of Natural Products (John Wiley & Sons, 2009).

[5] D. Oberthür , “Microcrystals in Structural Biology: Small Samples, Big Insights,” IUCrJ 12 (2025): 259–261, 10.1107/S2052252525003653. PMC12044849 40293197

[6] D. Shi , B. L. Nannenga , M. G. Iadanza , and T. Gonen , “Three‐Dimensional Electron Crystallography Of Protein Microcrystals,” Elife 2 (2013): 01345, 10.7554/eLife.01345. PMC3831942 24252878

[7] B. L. Nannenga , D. Shi , A. G. W. Leslie , and T. Gonen , “High‐Resolution Structure Determination By Continuous‐Rotation Data Collection In MicroED,” Nature Methods 11 (2014): 927–930, 10.1038/nmeth.3043. 25086503 PMC4149488

[8] C. G. Jones , M. W. Martynowycz , J. Hattne , et al., “The CryoEM Method MicroED as a Powerful Tool for Small Molecule Structure Determination,” ACS Central Science 4 (2018): 1587–1592, 10.1021/acscentsci.8b00760. 30555912 PMC6276044

[9] J. Unge , J. Lin , S. J. Weaver , A. Sae Her , and T. Gonen , “Compositional Analysis of Complex Mixtures using Automatic MicroED Data Collection,” Advanced Science 11 (2024): 2400081, 10.1002/advs.202400081. 38647272 PMC11187898

[10] M. J. de la Cruz , M. W. Martynowycz , J. Hattne , and T. Gonen , “MicroED Data Collection with SerialEM,” Ultramicroscopy 201 (2019): 77–80. 30986656 10.1016/j.ultramic.2019.03.009 PMC6752703

[11] J. Unge , B. L. Nannenga , A. G. Oliver , and T. Gonen , “Standards for MicroED,” Crystal Structure Communications 81 (2025): 376–390. 10.1107/S2053229625004875 PMC12231505 40539937

[12] L. J. Kim , M. Ohashi , Z. Zhang , et al., “Prospecting for Natural Products by Genome Mining and Microcrystal Electron Diffraction,” Nature Chemical Biology 17 (2021): 872–877, 10.1038/s41589-021-00834-2. 34312563 PMC8447837

[13] L. J. Kim , M. Xue , X. Li , et al., “Structure Revision of the Lomaiviticins,” Journal of the American Chemical Society 143 (2021): 6578–6585, 10.1021/jacs.1c01729. 33900077 PMC8935351

[14] J. D. Park , Y. Li , K. Moon , E. J. Han , S. R. Lee , and M. R. Seyedsayamdost , “Structural Elucidation of Cryptic Algaecides in Marine Algal‐Bacterial Symbioses by NMR Spectroscopy and MicroED,” Angewandte Chemie International Edition 61 (2022): 202114022, 10.1002/anie.202114022. PMC9056321 34852184

[15] T. E. Gorelik , K. H. M. E. Tehrani , T. Gruene , et al., “Crystal Structure Of Natural Product Argyrin‐D Determined by 3D Electron Diffraction,” CrystEngComm 24 (2022): 5885–5889, 10.1039/D2CE00707J.

[16] Y. Watanabe , S. Takahashi , S. Ito , et al., “Hakuhybotrol, A Polyketide Produced By Hypomyces Pseudocorticiicola , Characterized With the Assistance of 3D ED/MicroED,” Organic & Biomolecular Chemistry 21 (2023): 2320–2330, 10.1039/D2OB02286A. 36815714

[17] E. Danelius , G. Bu , L. H. E. Wieske , and T. Gonen , “MicroED as a Powerful Tool for Structure Determination of Macrocyclic Drug Compounds Directly From Their Powder Formulations,” ACS Chemical Biology 18 (2023): 2582–2589, 10.1021/acschembio.3c00611. 37944119 PMC10728894

[18] D. A. Delgadillo , L. Wu , C. Wang , et al., “Microcrystal Electron Diffraction‐Guided Discovery of Fungal Metabolites,” Journal of the American Chemical Society 147 (2025): 26158–26164, 10.1021/jacs.5c01466. 40460455 PMC12314910

[19] E. Danelius , S. Halaby , W. A. van der Donk , and T. Gonen , “MicroED in Natural Product And Small Molecule Research,” Natural Product Reports 38 (2021): 423–431, 10.1039/D0NP00035C. 32939523 PMC7965795

[20] D. A. Delgadillo , J. E. Burch , L. J. Kim , et al., “High‐Throughput Identification of Crystalline Natural Products From Crude Extracts Enabled by Microarray Technology and microED,” ACS Central Science 10 (2023): 176–183, 10.1021/acscentsci.3c01365. 38292598 PMC10823509

[21] J. Hattne , M. W. Martynowycz , P. A. Penczek , and T. Gonen , “MicroED With the Falcon III direct electron detector,” IUCrJ 6 (2019): 921–926, 10.1107/S2052252519010583. PMC6760445 31576224

[22] W. Kabsch , “xds,” Biological crystallography 66 (2010): 125–132. 20124692 10.1107/S0907444909047337 PMC2815665

[23] W. Kabsch , “Integration, Scaling, Space‐Group Assignment And Post‐Refinement,” Biological crystallography 66 (2010): 133–144. 20124693 10.1107/S0907444909047374 PMC2815666

[24] G. M. Sheldrick , “SHELXT—Integrated Space‐Group And Crystal‐Structure Determination,” Acta Crystallographica Section A Foundations and Advances 71 (2015): 3–8, 10.1107/S2053273314026370. 25537383 PMC4283466

[25] T. R. Schneider and G. M. Sheldrick , “Substructure Solution With SHELXD,” Acta Crystallographica Section D Biological Crystallography 58 (2002): 1772–1779, 10.1107/S0907444902011678. 12351820

[26] G. M. Sheldrick , “Crystal Structure Refinement with SHELXL,” Crystal Structure Communications 71 (2015): 3–8. 10.1107/S2053229614024218 PMC4294323 25567568

[27] F. Wessely and E. Demmer , “Dïe Konstitution des Fraxetins,” Berichte der deutschen chemischen Gesellschaft (A and B Series) 61 (1928): 1279–1284, 10.1002/cber.19280610616.

[28] P. T. Thuong , Y. R. Pokharel , M. Y. Lee , et al., “Dual Anti‐oxidative Effects of Fraxetin Isolated From Fraxinus rhinchophylla,” Biological and Pharmaceutical Bulletin 32 (2009): 1527–1532, 10.1248/bpb.32.1527. 19721227

[29] N. M. Ha and N. T. Son , “Health benefits of fraxetin: From chemistry to medicine,” Archiv der Pharmazie 357 (2024): 2400092, 10.1002/ardp.202400092. 38501886

[30] K. C. Huang , The Pharmacology of Chinese Herbs (CRC Press, 1998).

[31] L. C. Di Stasi , “Coumarin Derivatives in Inflammatory Bowel Disease,” Molecules (Basel, Switzerland) 26 (2021): 422, 10.3390/molecules26020422. 33467396 PMC7830946

[32] M. Rossi , S. Aktar , M. Davis , et al., “The Grapefruit Effect: Interaction Between Cytochrome P450 and Coumarin Food Components, Bergamottin, Fraxidin and Osthole. X‐ray Crystal Structure and DFT Studies,” Molecules (Basel, Switzerland) 25 (2020): 3158, 10.3390/molecules25143158. 32664320 PMC7397038

[33] C. Li , B.‐S. Luo , and T.‐E. Sun , “Molecular and Crystal Structure of 7‐Hydroxy‐6,8‐dimethoxyl‐coumarin,” Chinese Journal of Structural Chemistry 20 (2001): 100.

[34] T. Saitoh and S. Shibata , “New Type Of Chalcones From Licorice Root,” Tetrahedron Letters 50 (1975): 4461–4462.

[35] Y. Fu , T. Hsieh , J. Guo , et al., “Licochalcone‐A, A Novel Flavonoid Isolated From Licorice Root (Glycyrrhiza glabra), Causes G2 and Late‐G1 Arrests In Androgen‐Independent PC‐3 Prostate Cancer Cells,” Biochemical and Biophysical Research Communications 322 (2004): 263–270, 10.1016/j.bbrc.2004.07.094. 15313200

[36] M. Liu , Y. Du , and D. Gao , “Licochalcone A: A Review Of Its Pharmacology Activities And Molecular Mechanisms,” Frontiers in Pharmacology 15 (2024): 1453426, 10.3389/fphar.2024.1453426. 39188947 PMC11345200

[37] M.‐T. Li , L. Xie , H.‐M. Jiang , et al., “Role of Licochalcone A in Potential Pharmacological Therapy: A Review,” Frontiers in Pharmacology 13 (2022): 878776, 10.3389/fphar.2022.878776. 35677438 PMC9168596

[38] Z. Rozmer and P. Perjési , “Naturally Occurring Chalcones And Their Biological Activities,” Phytochemistry Reviews 15 (2016): 87–120, 10.1007/s11101-014-9387-8.

[39] S. Shaikh , E. J. Lee , K. Ahmad , and I. Choi , “Therapeutic Potential And Action Mechanisms Of Licochalcone B: A Mini Review,” Frontiers in Molecular Biosciences 11 (2024): 1440132, 10.3389/fmolb.2024.1440132. 39021879 PMC11251949

[40] J.‐S. Liu , Y.‐L. Zhu , C.‐M. Yu , et al., “The Structures Of Huperzine A and B, Two New Alkaloids Exhibiting Marked Anticholinesterase Activity,” Canadian Journal of Chemistry 64 (1986): 837–839, 10.1139/v86-137.

[41] D. Bai , “Development of Huperzine A and B for Treatment of Alzheimer's Disease,” Pure and Applied Chemistry 79 (2007): 469–479, 10.1351/pac200779040469.

[42] H. Y. Zhang and X. C. Tang , “Huperzine B, A Novel Acetylcholinesterase Inhibitor, Attenuates Hydrogen Peroxide Induced Injury in PC12 Cells,” Neuroscience Letters 292 (2000): 41–44, 10.1016/S0304-3940(00)01433-6. 10996445

[43] S. Jiang , B.‐B. Gao , Y.‐F. Ou , and Q.‐S. Zhao , “Lycopodium alkaloids From Huperzia serrata and their cholinesterase inhibitory activities,” Phytochemistry 223 (2024): 114114, 10.1016/j.phytochem.2024.114114. 38697240

[44] J.‐M. Jiang , D. Xia , X.‐L. Zhu , D. Zhu , X.‐W. Yang , and K. Pan , “Lycophlegmarinines A–F, new Lycopodium alkaloids From Phlegmariurus phlegmaria,” Tetrahedron 114 (2022): 132782, 10.1016/j.tet.2022.132782.

[45] H. Dvir , H. L. Jiang , D. M. Wong , et al., “X‐ray Structures of Torpedo californica Acetylcholinesterase Complexed With (+)‐Huperzine A and (−)‐Huperzine B: Structural Evidence for an Active Site Rearrangement,” Biochemistry 41 (2002): 10810–10818, 10.1021/bi020151+. 12196020

[46] J. B. Koepfli , J. A. Brockman Jr , and J. Moffat , “The Structure Of Febrifugine And Isofebrifugine,” Journal of the American Chemical Society 72 (1950): 3323–3323.

[47] G. Rukunga and A. J. Simons , The Potential of Plants as a Source of Antimalarial Agents: A Review (PlantaPhile Publications, 2006): pp. 187–206.

[48] S. S. Ningthoujam , A. D. Talukdar , D. Nath , N. Basar , K. S. Potsangbam , and M. D. Choudhury , “Febrifugine and Its Analogs: Studies for Their Antimalarial And Other Therapeutic Properties,” Studies in natural products chemistry 44 (2015): 93–112.

[49] N. P. McLaughlin , P. Evans , and M. Pines , “The Chemistry And Biology Of Febrifugine And Halofuginone,” Bioorganic & Medicinal Chemistry 22 (2014): 1993–2004, 10.1016/j.bmc.2014.02.040. 24650700

[50] S. Kobayashi , M. Ueno , R. Suzuki , H. Ishitani , H.‐S. Kim , and Y. Wataya , “Catalytic Asymmetric Synthesis of Antimalarial Alkaloids Febrifugine and Isofebrifugine and Their Biological Activity,” The Journal of Organic Chemistry 64 (1999): 6833–6841, 10.1021/jo990877k. 11674693

[51] S. Kobayashi , M. Ueno , R. Suzuki , and H. Ishitani , “Catalytic Asymmetric Synthesis of Febrifugine And Isofebrifugine,” Tetrahedron Letters 40 (1999): 2175–2178, 10.1016/S0040-4039(99)00142-2. 11674693

[52] T. M. M. Maiden , N. Mbelesi , P. A. Procopiou , S. Swanson , and J. P. A. Harrity , “A Convergent Strategy Towards Febrifugine And Related Compounds,” Organic & Biomolecular Chemistry 16 (2018): 4159–4169, 10.1039/C8OB00935J. 29786725

[53] F. Li and Y. L. Liu , “bao zuo zuo ‐ I,VI,VII he bao zuo su de fen li he jie gou yan jiu (宝藿甙‐I,VI,VII,和宝藿素的分离和结构研究),” Acta pharmaceutica Sinica 23 (1988): 739–748. 3257026

[54] J. A. Duke , Handbook of Phytochemical Constituent Grass, Herbs and Other Economic Plants: Herbal Reference Library (Routledge, 2017), 10.1201/9780203752623.

[55] J. Mu , Y. Li , Q. Chen , et al., “Revealing the Molecular Mechanism Of Baohuoside I for the Treatment Of Breast Cancer Based On Network Pharmacology And Molecular Docking,” Journal of Ethnopharmacology 337 (2025): 118918, 10.1016/j.jep.2024.118918. 39396715

[56] X. Zhang , B. Tang , S. Wen , et al., “Advancements in the Biotransformation and Biosynthesis of the Primary Active Flavonoids Derived From Epimedium,” Molecules (Basel, Switzerland) 28 (2023): 7173, 10.3390/molecules28207173. 37894651 PMC10609448

[57] L. Jia , Q. Zhang , J.‐R. Wang , and X. Mei , “Versatile solid modifications of icariin: Structure, properties and form transformation,” CrystEngComm 17 (2015): 7500–7509, 10.1039/C5CE01422K.

[58] D. Cremer and J. A. Pople , “General Definition Of Ring Puckering Coordinates,” Journal of the American Chemical Society 97 (1975): 1354–1358, 10.1021/ja00839a011.

[59] J. Polonsky , Z. Baskévitch , A. Gaudemer , and B. C. Das , “Constituants amers deBrucea amarissima structures des brucéines A, B et C,” Experientia 23 (1967): 424–426, 10.1007/BF02142154. 4168866

[60] J. Zhang , H.‐X. Xu , Y.‐X. Dou , Q.‐H. Huang , Y.‐F. Xian , and Z.‐X. Lin , “Major Constituents From Brucea javanica and Their Pharmacological Actions,” Frontiers in Pharmacology 13 (2022): 853119, 10.3389/fphar.2022.853119. 35370639 PMC8971814

[61] B.‐N. Su , L. C. Chang , E. J. Park , et al., “Bioactive Constituents of the Seeds of Brucea javanica,” Planta Medica 68 (2002): 730–733, 10.1055/s-2002-33798. 12221597

[62] K. L. Chan , S. Lee , T. W. Sam , and B. H. Han , “A Quassinoid Glycoside From the Roots of Eurycoma longifolia,” Phytochemistry 28 (1989): 2857–2859, 10.1016/S0031-9422(00)98108-1.

[63] C. H. Teh , S. C. Teoh , C. S. Yeap , K. L. Chan , and H. K. Fun , “4, 5, 7, 8, 17‐Pentahydroxy‐14, 18‐dimethyl‐6‐methylene‐3, 10‐dioxapentacyclo [9.8. 0.01, 7.04, 19.013, 18] nonadec‐14‐ene‐9, 16‐dione methanol solvate dihydrate,” Structure Reports 65 (2009): o898—o899. 10.1107/S1600536809010502 PMC2968991 21582604

[64] H. Naora , M. Ishibashi , T. Furuno , et al., “Structure Determination of Bitter Principles in Ailanthus altissima. Structure of Shinjulactone A and Revised Structure of Ailanthone,” Bulletin of the Chemical Society of Japan 56 (1983): 3694–3698, 10.1246/bcsj.56.3694.

[65] C.‐H. Teh , M. Abdulghani , H. Morita , M. Shiro , A. H. Hussin , and K.‐L. Chan , “Comparative X‐Ray and Conformational Analysis of a New Crystal of 13 α ,21‐Dihydroeurycomanone With Eurycomanone From Eurycoma longifolia and Their Anti‐Estrogenic Activity Using the Uterotrophic Assay,” Planta Medica 77 (2011): 128–132, 10.1055/s-0030-1250159. 20665368

[66] D. Lavie and M. K. Jain , “Tetranortriterpenoids from Melia azadirachta L.,” Chemical Communications (1967): 278–280.

[67] T. Akihisa , J. Zhang , A. Manosroi , T. Kikuchi , J. Manosroi , and M. Abe , “Limonoids and Other Secondary Metabolites of Azadirachta indica (neem) and Azadirachta indica var. siamensis (Siamese neem), and Their Bioactivities,” Studies in Natural Products Chemistry 68 (Elsevier, 2021): pp. 29–65.

[68] R. Sakib , F. Caruso , S. Belli , and M. Rossi , “Azadiradione, a Component of Neem Oil, Behaves as a Superoxide Dismutase Mimic When Scavenging the Superoxide Radical, as Shown Using DFT and Hydrodynamic Voltammetry,” Biomedicines 11 (2023): 3091, 10.3390/biomedicines11113091. 38002091 PMC10669394

[69] Y.‐S. Shi , Y. Zhang , H.‐T. Li , et al., “Limonoids From Citrus: Chemistry, anti‐tumor potential, and other bioactivities,” Journal of Functional Foods 75 (2020): 104213, 10.1016/j.jff.2020.104213.

[70] R. Malathi , S. S. Rajan , M. S. Krishnan , G. Gopalakrishnan , and G. Suresh , “Azadirone,” Structure Reports 62 (2006): o5694—o5696.

[71] S. Sarkhel , G. K. Jain , S. Singh , H. S. Subramanya , and P. R. Maulik , “Dysobinin, A Tetranortriterpenoid,” Crystal Structure Communications 56 (2000): iuc0000117–e254. 10.1107/S0108270100006041 15263115

