# Supplementary material for: Rapid Structural Analysis of Natural Products Using MicroED
Source: Small. 2026 Jan 23;22(15):e11875. doi: 10.1002/smll.202511875 (PMC12980483; doi:10.1002/smll.202511875)
Supplement: Supplementary file 1 — Supporting File: smll72428‐sup‐0001‐SuppMat.docx. [file SMLL-22-e11875-s001.docx]

**Supporting Information**

**Rapid structural analysis of natural products using MicroED**

Jieye Lin^1,6^, Orel Paz^1,2,3,6^, Johan Unge^4^ and Tamir Gonen^1,2,3,5^*

^1^ Department of Biological Chemistry, University of California, Los Angeles, 615 Charles E. Young Drive South, Los Angeles, California 90095, United States

^2^ Molecular Biology Institute, University of California, Los Angeles, Los Angeles, CA 90095, USA.

^3^ Howard Hughes Medical Institute, University of California, Los Angeles, Los Angeles, California 90095, United States

^4^ Department of Chemistry, Umeå University, 901 87 Umeå, Sweden

^5^ Department of Physiology, University of California, Los Angeles, 615 Charles E. Young Drive South, Los Angeles, California 90095, United States

^6^ These authors contributed equally to this work.

^*^ Corresponding Author T.G. tgonen@g.ucla.edu


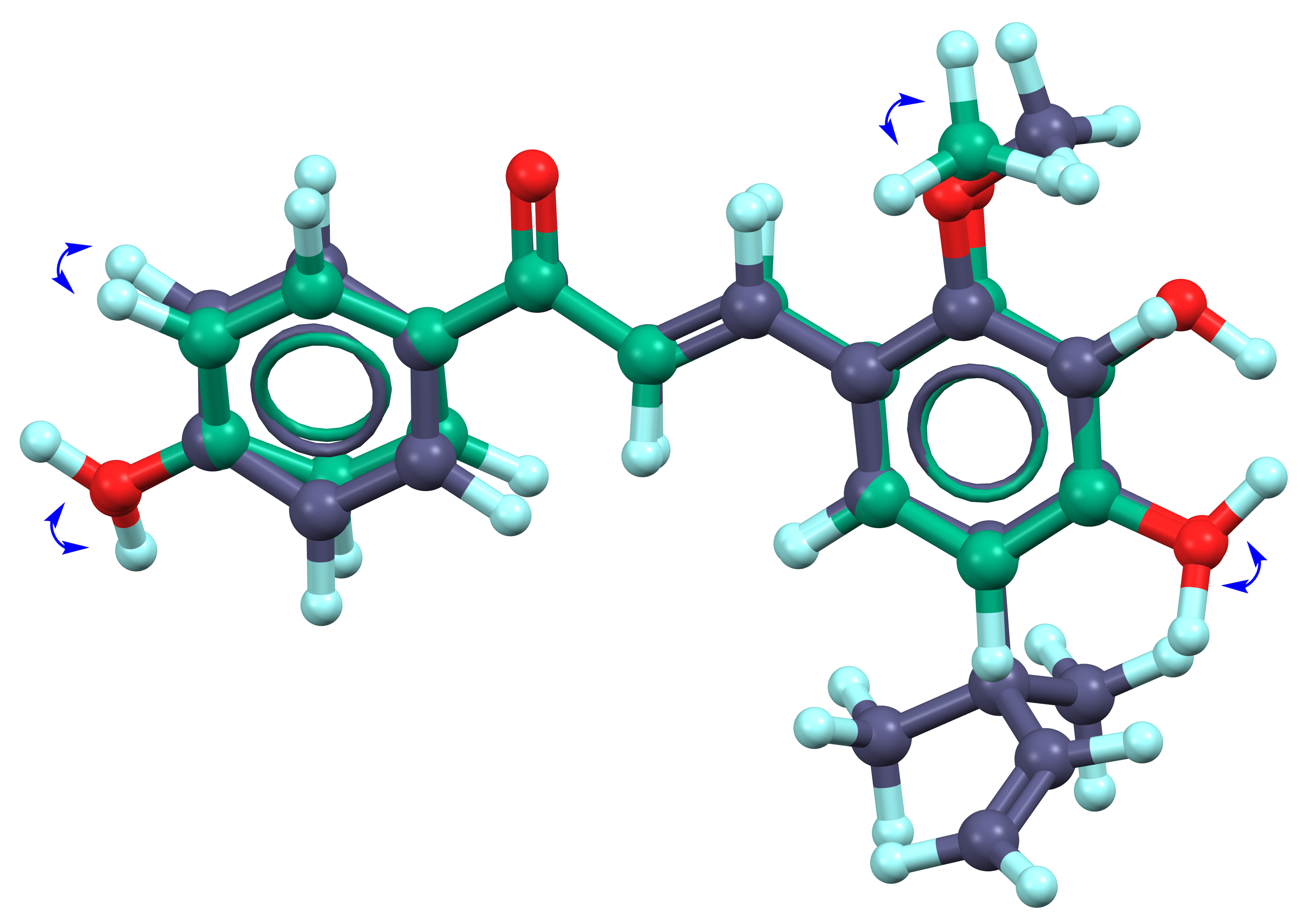


**Figure S1** Overlaying MicroED structures of **2** (form 1) and **3** showing major torsional changes in the phenyl and exocyclic groups. **2** (form 1) is presented in purple, and **3** is presented in green.

**
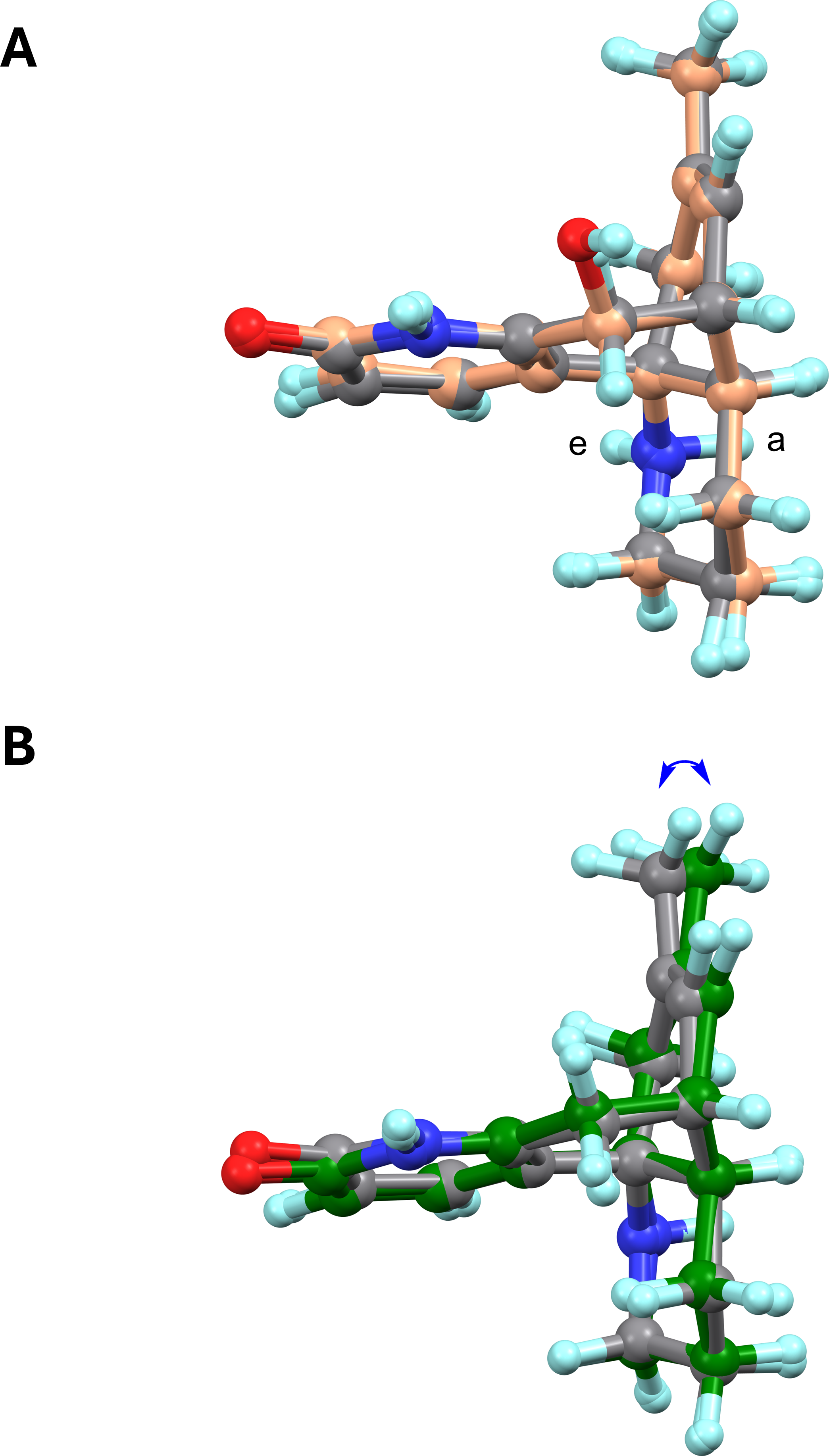
**

**Figure S2** (A) Overlaying MicroED structure **4** and huperradine A as determined with X-ray crystallography (CCDC entry: JOCTEE)^1^ showing comparable backbone geometry. (B) Overlaying MicroED structure **4** and its protein-bound conformation extracted from PDB structure 1GPN,^2^ showing the structural differences.


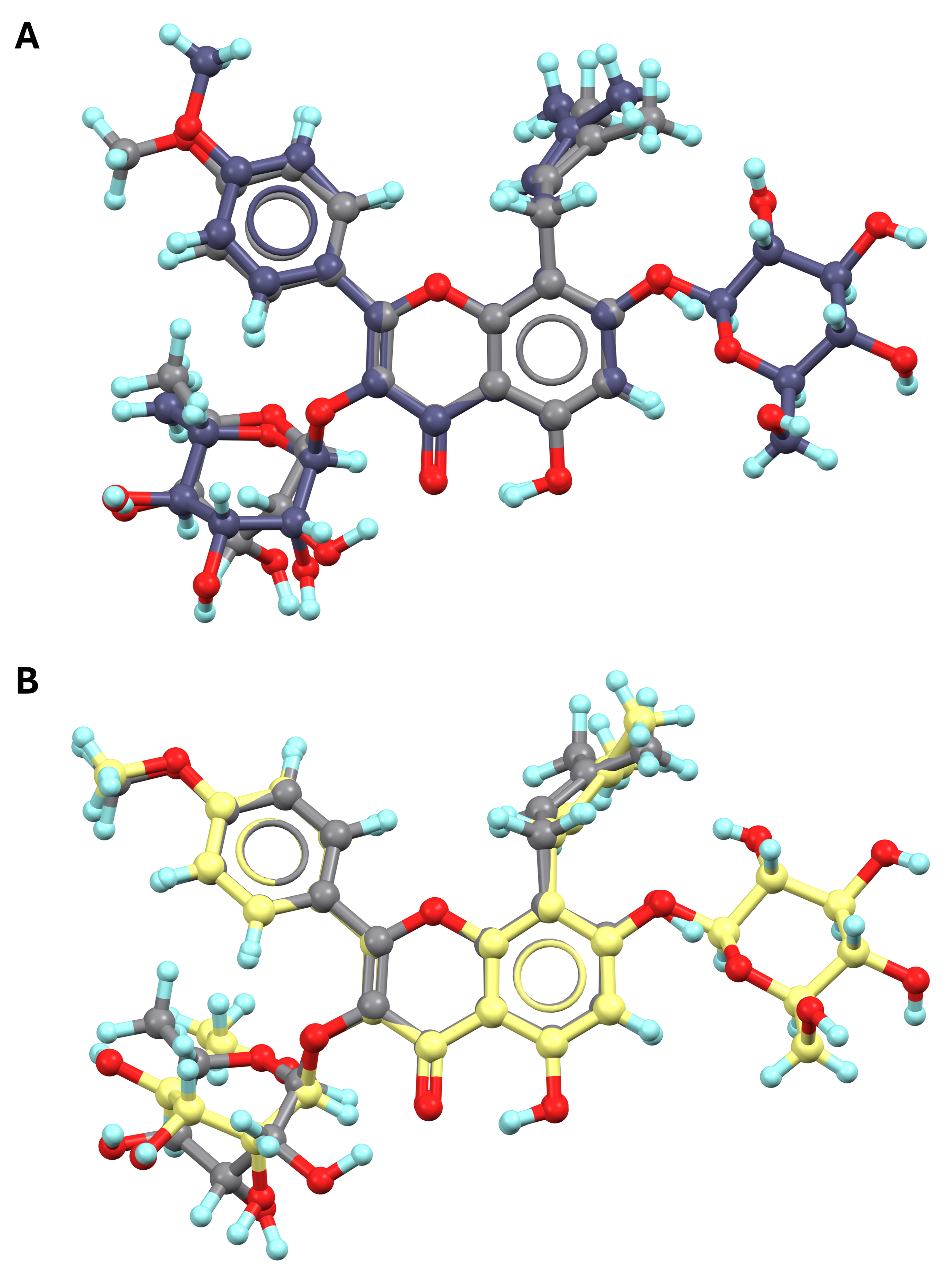


**Figure S3** (A) Overlaying MicroED structure **6** and icarrin form 1 of the deposited X-ray crystallographic structure (CCDC entry: KUNPAM);^3^ (B) Overlaying MicroED structure **6** and icarrin form 2 (CCDC entry: KUNPAM).^3^ **6** was colored in grey, Icariin forms 1 and 2 were colored in purple and yellow, respectively.


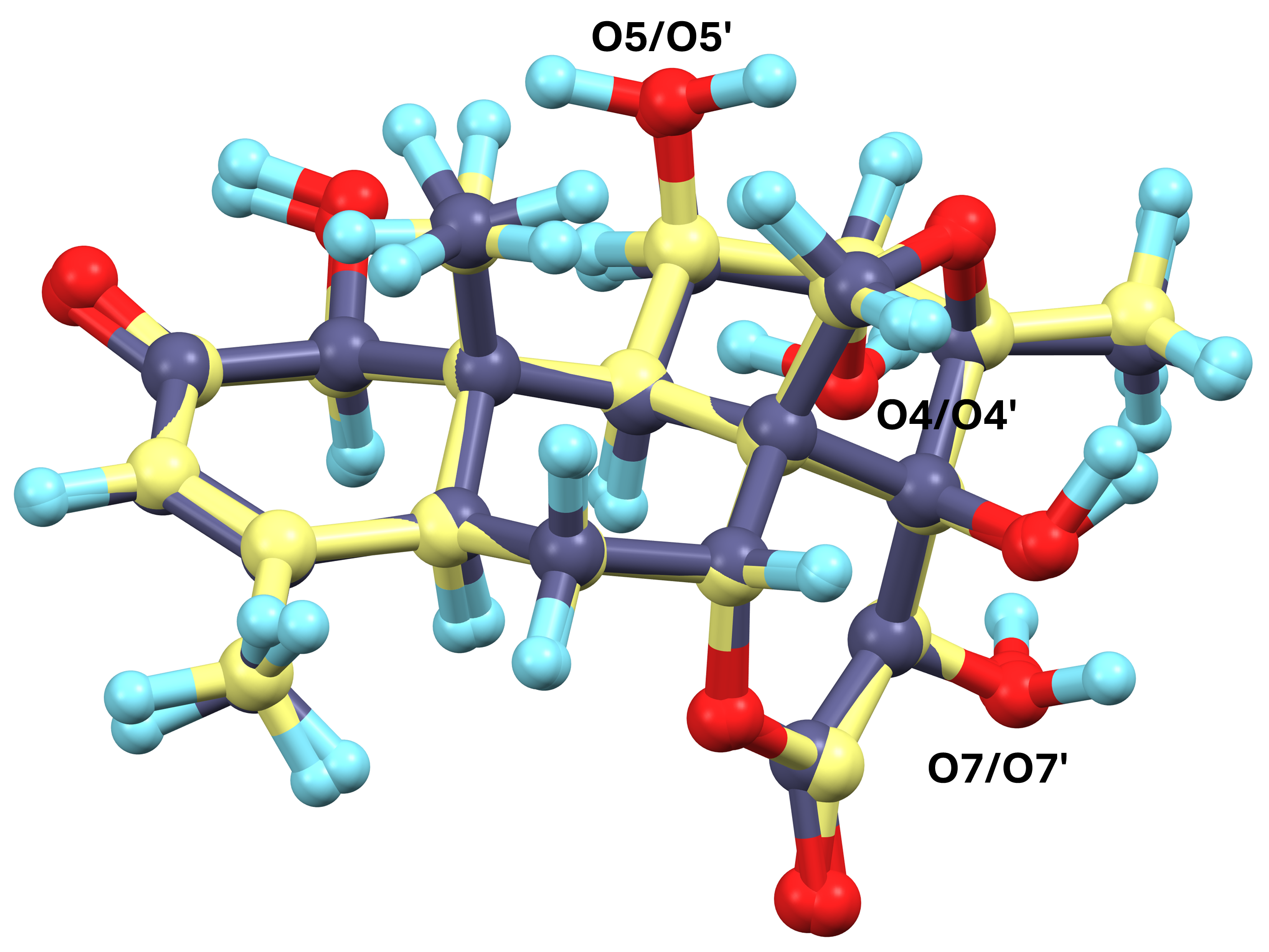


**Figure S4** Overlaying polymorphic MicroED structures of **7** showing the structural differences in exocyclic hydroxyl groups and rings. Form 1 was presented in purple, form 2 was presented in yellow.

**
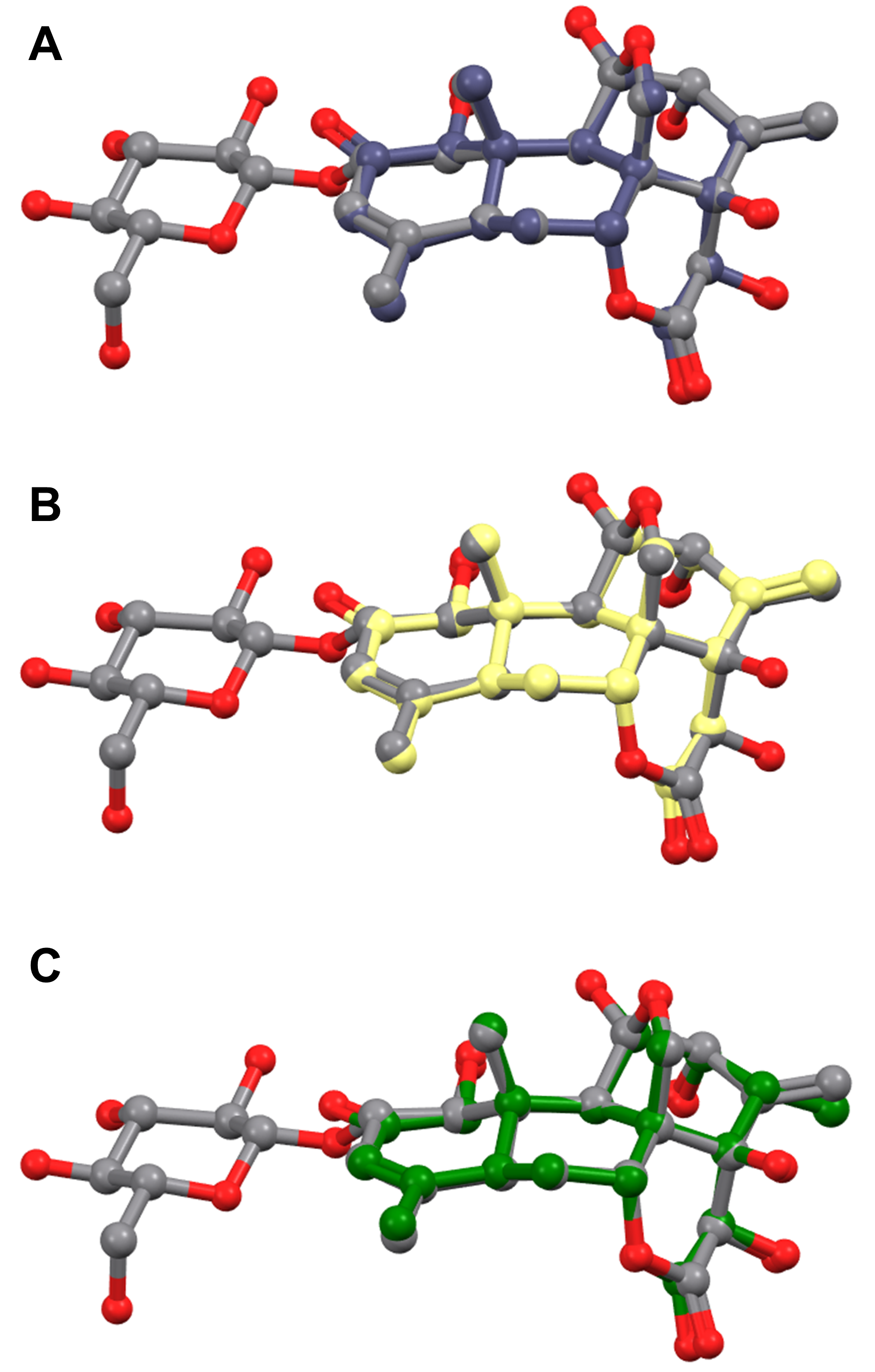
**

**Figure S5** (A) Overlay structures of **8** and eurycomanone (CCDC entry: VOQPIB);^4^ (B) Overlay structures if **8** and ailanthone (BITWIK10);^5^ (C) Overlay structures **8** and pasakbumin C (EZISUC).^6^ **8**, eurycomanone, ailanthone and pasakbumin were presented in grey, purple, yellow and green, respectively.


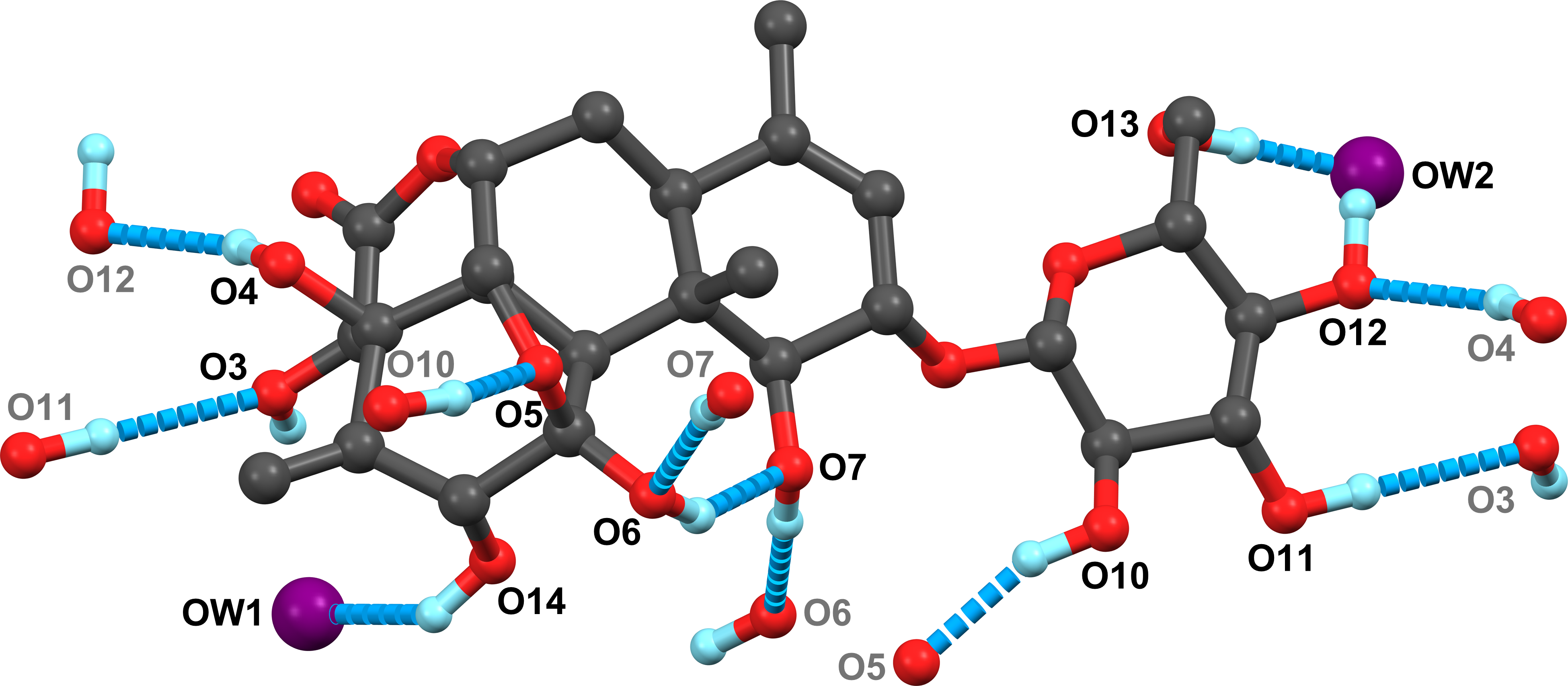


**Figure S6** Hydrogen bonding interactions in **8**. Hydrogen bonding interactions were presented in blue dashed lines. Water molecules were colored in purple balls.


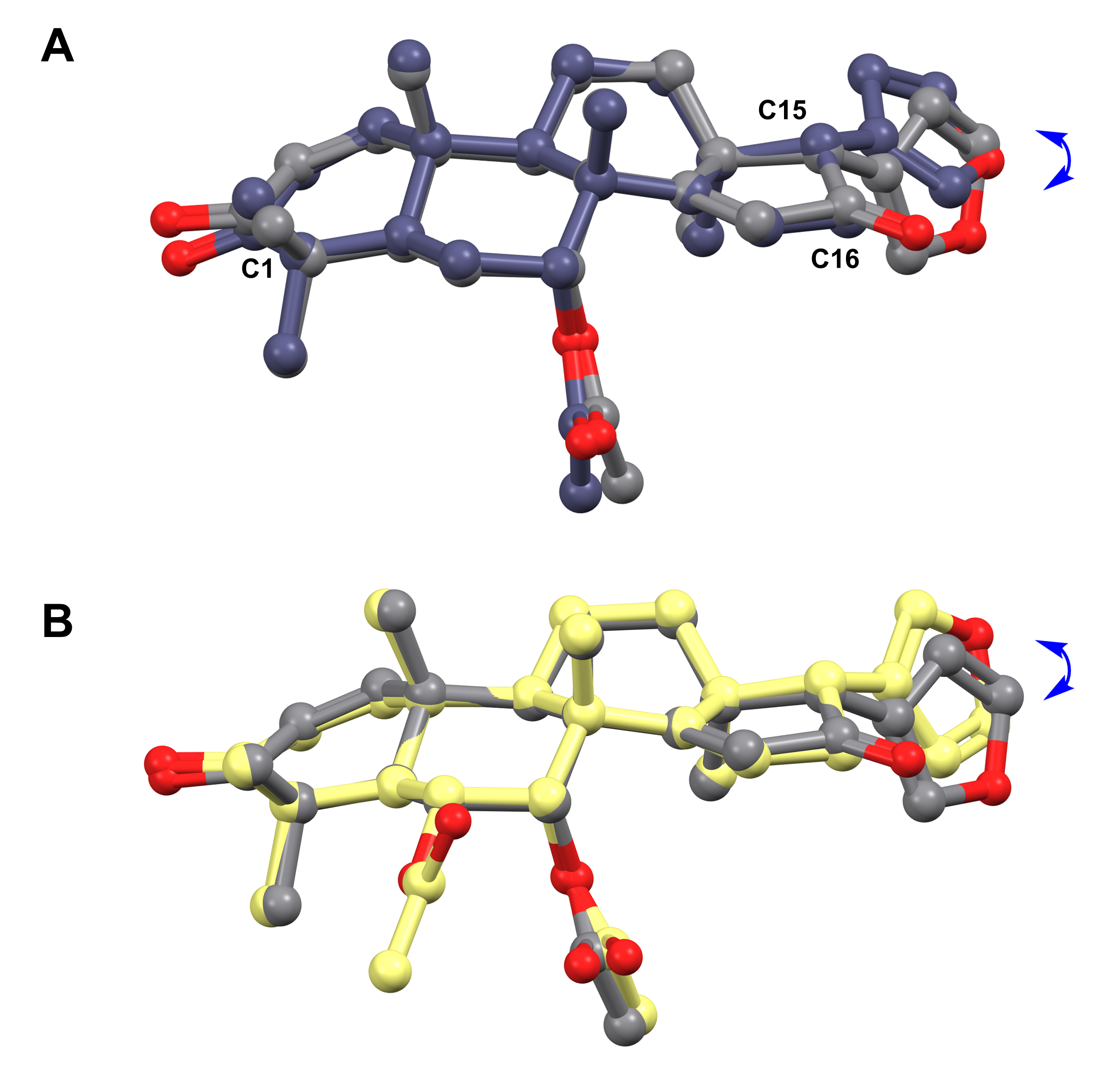


**Figure S7** (A) Overlay structures of **9** and azadirone (CCDC entry: TERNAG);^7^ (B) Overlay structures of **9** and 6β-acetoxyazadirone (LIYYIB).^8^ **9**, azadirone and 6β-acetoxyazadirone were presented in grey, purple and yellow, respectively.

**
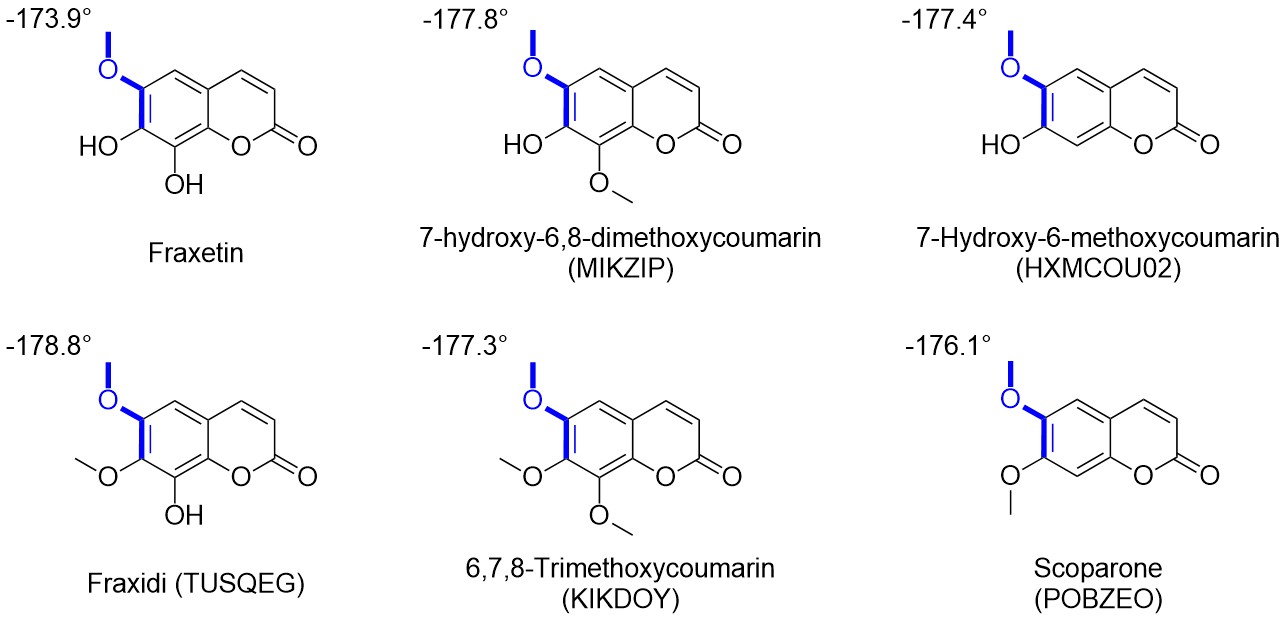
**

**Scheme S1** Chemical structures of fraxetin (**1**) and related compounds.^9-13^ The ‒OCH_3_ groups (colored in blue) positioned away from proximal ‒OH or ‒OCH_3_ group as measured from their crystal structures.

**Table S1** MicroED data statistics of Fraxetin (**1**).

| Stoichiometric formula | C_10_ H_8_ O_5_ |
| --- | --- |
| Molar mass | 208.16 |
| Temperature (K) | 80 |
| Crystal system | Orthorhombic |
| Space group | *F*dd2 |
| Unit cell lengths (Å) |  |
| a | 17.480 |
| b | 27.380 |
| c | 6.930 |
| Unit cell angles (°) |  |
| α | 90.00 |
| β | 90.00 |
| γ | 90.00 |
| Cell volume (Å^3^) | 3316.7 |
| No. of merged datasets | 2 |
| No. of observed reflections | 15995 |
| No. of unique reflections | 1918 |
| R_obs_ (%) | 34.9 |
| R_meas_ (%) | 37.2 |
| I/Sigma | 5.66 |
| CC_1/2_ | 98.7 |
| **Resolution (Å)** | **0.63** |
| **Completeness (%)** | **99.3** |
| **R_1_ (%)** | **13.25** |
| *w*R_2_ (%) | 34.62 |
| GooF | 1.098 |

**Table S2** MicroED data statistics of Licochalcone A (**2**).

| Stoichiometric formula | C_21_ H_22_ O_4_ |
| --- | --- |
| Molar mass | 338.39 |
| Temperature (K) | 80 |
| Crystal system | Triclinic |
| Space group | *P*-1 |
| Unit cell lengths (Å) |  |
| a | 11.490 |
| b | 12.390 |
| c | 13.660 |
| Unit cell angles (°) |  |
| α | 75.10 |
| β | 77.61 |
| γ | 66.13 |
| Cell volume (Å^3^) | 1705.0 |
| No. of merged datasets | 9 |
| No. of observed reflections | 76231 |
| No. of unique reflections | 7579 |
| R_obs_ (%) | 39.9 |
| R_meas_ (%) | 42.1 |
| I/Sigma | 4.37 |
| CC_1/2_ | 96.3 |
| **Resolution (Å)** | **0.75** |
| **Completeness (%)** | **89.1** |
| **R_1_ (%)** | **16.95** |
| *w*R_2_ (%) | 45.55 |
| GooF | 1.183 |

**Table S3** MicroED data statistics of Licochalcone B (**3**).

| Stoichiometric formula | C_16_ H_14_ O_5_ |
| --- | --- |
| Molar mass | 286.27 |
| Temperature (K) | 80 |
| Crystal system | Monoclinic |
| Space group | *P*2_1_/c |
| Unit cell lengths (Å) |  |
| a | 7.780 |
| b | 10.310 |
| c | 16.180 |
| Unit cell angles (°) |  |
| α | 90.00 |
| β | 102.51 |
| γ | 90.00 |
| Cell volume (Å^3^) | 1267.0 |
| No. of merged datasets | 2 |
| No. of observed reflections | 17544 |
| No. of unique reflections | 3932 |
| R_obs_ (%) | 29.2 |
| R_meas_ (%) | 33.2 |
| I/Sigma | 4.20 |
| CC_1/2_ | 97.9 |
| **Resolution (Å)** | **0.70** |
| **Completeness (%)** | **96.8** |
| **R_1_ (%)** | **17.20** |
| *w*R_2_ (%) | 44.64 |
| GooF | 1.139 |

**Table S4** MicroED data statistics of Huperzine B (**4**).

| Stoichiometric formula | C_16_ H_20_ N_2_ O |
| --- | --- |
| Molar mass | 256.34 |
| Temperature (K) | 80 |
| Crystal system | Orthorhombic |
| Space group | *P*2_1_2_1_2_1_ |
| Unit cell lengths (Å) |  |
| a | 7.400 |
| b | 11.880 |
| c | 14.520 |
| Unit cell angles (°) |  |
| α | 90.0 |
| β | 90.0 |
| γ | 90.0 |
| Cell volume (Å^3^) | 1276.5 |
| No. of merged datasets | 1 |
| No. of observed reflections | 9805 |
| No. of unique reflections | 2382 |
| R_obs_ (%) | 20.5 |
| R_meas_ (%) | 23.6 |
| I/Sigma | 4.26 |
| CC_1/2_ | 96.8 |
| **Resolution (Å)** | **0.67** |
| **Completeness (%)** | **93.5** |
| **R_1_ (%)** | **16.04** |
| *w*R_2_ (%) | 40.50 |
| GooF | 1.095 |

**Table S5** MicroED data statistics of Isofebrifugine (**5**).

| Stoichiometric formula | C_16_ H_19_ N_3_ O_3_ |
| --- | --- |
| Molar mass | 301.34 |
| Temperature (K) | 80 |
| Crystal system | Monoclinic |
| Space group | *P*2_1_ |
| Unit cell lengths (Å) |  |
| a | 8.320 |
| b | 6.430 |
| c | 13.520 |
| Unit cell angles (°) |  |
| α | 90.0 |
| β | 104.7 |
| γ | 90.0 |
| Cell volume (Å^3^) | 699.5 |
| No. of merged datasets | 2 |
| No. of observed reflections | 17717 |
| No. of unique reflections | 3491 |
| R_obs_ (%) | 20.8 |
| R_meas_ (%) | 23.4 |
| I/Sigma | 5.79 |
| CC_1/2_ | 98.0 |
| **Resolution (Å)** | **0.60** |
| **Completeness (%)** | **95.9** |
| **R_1_ (%)** | **13.89** |
| *w*R_2_ (%) | 39.41 |
| GooF | 1.079 |

**Table S6** MicroED data statistics of Baohuoside I (**6**).

| Stoichiometric formula | C_27_ H_30_ O_10_ |
| --- | --- |
| Molar mass | 514.51 |
| Temperature (K) | 80 |
| Crystal system | Monoclinic |
| Space group | *P*2_1_ |
| Unit cell lengths (Å) |  |
| a | 13.190 |
| b | 4.680 |
| c | 19.890 |
| Unit cell angles (°) |  |
| α | 90.0 |
| β | 93.4 |
| γ | 90.0 |
| Cell volume (Å^3^) | 1225.6 |
| No. of merged datasets | 4 |
| No. of observed reflections | 28551 |
| No. of unique reflections | 3537 |
| R_obs_ (%) | 18.7 |
| R_meas_ (%) | 19.9 |
| I/Sigma | 7.27 |
| CC_1/2_ | 99.3 |
| **Resolution (Å)** | **0.73** |
| **Completeness (%)** | **95.8** |
| **R_1_ (%)** | **12.23** |
| *w*R_2_ (%) | 34.03 |
| GooF | 1.080 |

**Table S7** MicroED data statistics of Bruceine D (**7**).

| Stoichiometric formula | C_20_ H_26_ O_9_ |
| --- | --- |
| Molar mass | 410.41 |
| Temperature (K) | 80 |
| Crystal system | Monoclinic |
| Space group | *P*2_1_ |
| Unit cell lengths (Å) |  |
| a | 6.490 |
| b | 19.890 |
| c | 13.840 |
| Unit cell angles (°) |  |
| α | 90.0 |
| β | 91.3 |
| γ | 90.0 |
| Cell volume (Å^3^) | 1786.1 |
| No. of merged datasets | 5 |
| No. of observed reflections | 41164 |
| No. of unique reflections | 4060 |
| R_obs_ (%) | 27.3 |
| R_meas_ (%) | 28.8 |
| I/Sigma | 7.35 |
| CC_1/2_ | 98.2 |
| **Resolution (Å)** | **0.78** |
| **Completeness (%)** | **100.0** |
| **R_1_ (%)** | **17.85** |
| *w*R_2_ (%) | 43.69 |
| GooF | 1.156 |

**Table S8** MicroED data statistics of Eurycomanol 2-O-β-D-glucopyranoside dihydrate (**8**).

| Stoichiometric formula | C_26_ H_36_ O_14_, 2 O |
| --- | --- |
| Molar mass | 604.55 |
| Temperature (K) | 80 |
| Crystal system | Monoclinic |
| Space group | *P*2_1_ |
| Unit cell lengths (Å) |  |
| a | 8.520 |
| b | 9.550 |
| c | 15.790 |
| Unit cell angles (°) |  |
| α | 90.0 |
| β | 95.0 |
| γ | 90.0 |
| Cell volume (Å^3^) | 1279.8 |
| No. of merged datasets | 3 |
| No. of observed reflections | 24357 |
| No. of unique reflections | 3109 |
| R_obs_ (%) | 18.5 |
| R_meas_ (%) | 19.9 |
| I/Sigma | 7.78 |
| CC_1/2_ | 98.7 |
| **Resolution (Å)** | **0.73** |
| **Completeness (%)** | **85.1** |
| **R_1_ (%)** | **17.28** |
| *w*R_2_ (%) | 47.37 |
| GooF | 1.653 |

**Table S9** MicroED data statistics of Azadiradione (**9**).

| Stoichiometric formula | C_28_ H_34_ O_5_ |
| --- | --- |
| Molar mass | 450.55 |
| Temperature (K) | 80 |
| Crystal system | Tetragonal |
| Space group | *P*4_3_2_1_2 |
| Unit cell lengths (Å) |  |
| a | 15.820 |
| b | 15.820 |
| c | 18.140 |
| Unit cell angles (°) |  |
| α | 90.0 |
| β | 90.0 |
| γ | 90.0 |
| Cell volume (Å^3^) | 4539.9 |
| No. of merged datasets | 1 |
| No. of observed reflections | 18404 |
| No. of unique reflections | 2164 |
| R_obs_ (%) | 27.4 |
| R_meas_ (%) | 29.2 |
| I/Sigma | 6.12 |
| CC_1/2_ | 98.3 |
| **Resolution (Å)** | **0.86** |
| **Completeness (%)** | **98.1** |
| **R_1_ (%)** | **13.47** |
| *w*R_2_ (%) | 32.94 |
| GooF | 1.174 |

**Reference:**

1. S. Jiang, B.-B. Gao, Y.-F. Ou, Q.-S. Zhao, *Phytochemistry* **2024**, *223*, 114114.
2. H. Dvir, H. L. Jiang, D. M. Wong, M. Harel, M. Chetrit, X. C. He, G. Y. Jin, G. L. Yu, X. C. Tang, I. Silman, *Biochemistry* **2002**, *41*, 10810-10818.
3. L. Jia, Q. Zhang, J.-R. Wang, X. Mei, *CrystEngComm* **2015**, *17*, 7500-7509.
4. C. H. Teh, S. C. Teoh, C. S. Yeap, K. L. Chan, H. K. Fun, *Structure Reports* **2009**, *65*, o898-o899.
5. H. Naora, M. Ishibashi, T. Furuno, T. Tsuyuki, T. Murae, H. Hirota, T. Takahashi, A. Itai, Y. Iitaka, *Bulletin of the Chemical Society of Japan* **1983**, *56*, 3694-3698.
6. C.-H. Teh, M. Abdulghani, H. Morita, M. Shiro, A. H. Hussin, K.-L. Chan, *Planta medica* **2011**, *77*, 128-132.
7. R. Malathi, S. S. Rajan, M. S. Krishnan, G. Gopalakrishnan, G. Suresh, *Structure Reports* **2006**, *62*, o5694-o5696.
8. S. Sarkhel, G. K. Jain, S. Singh, H. S. Subramanya, P. R. Maulik, *Crystal Structure Communications* **2000**, *56*, iuc0000117-e0000254.
9. M. Rossi, S. Aktar, M. Davis, E. Hefter Feuss, S. Roman-Holba, K. Wen, C. Gahn, F. Caruso, *Molecules* **2020**, *25*, 3158.
10. G. M. Happi, G. P. M. Kemayou, H.-G. Stammler, B. Neumann, M. Ismail, S. F. Kouam, J. D. Wansi, J. C. Tchouankeu, M. Frese, B. N. Lenta, *Phytochemistry* **2021**, *181*, 112537.
11. Li, C.; Luo, B.-S.; Sun, T.-E. *Jiegou Huaxue* **2001**, *20*, 100.
12. N. Saidi, M. R. Mukhtar, K. Awang, A. H. A. Hadi, S. W. Ng, *Structure Reports* **2007**, *63*, o3692-o3693.
13. J.-y. Chen, X.-y. Wang, M.-h. Hong, D.-x. Yi, M.-h. Qi, G.-b. Ren, *Journal of Molecular Structure* **2019**, *1191*, 323-336.
